# Baseline and placebo-related imaging, cerebrospinal fluid, plasma biomarker, and cognitive findings in unimpaired *PSEN1 E280A* mutation carriers and non-carriers in the Alzheimer’s Prevention Initiative Autosomal Dominant Alzheimer’s Disease Colombia Trial

**DOI:** 10.1016/j.ebiom.2026.106390

**Published:** 2026-07-17

**Authors:** Tobias Bittner, Francisco Lopera, Silvia Rios-Romenets, Courtney Schiffman, David Clayton, Derrek Hibar, Gwendlyn Kollmorgen, Michael Dolton, Victor Poon, Jonas Nguyen, Margarita Giraldo-Chica, Natalia Acosta-Baena, Alejandro Espinosa, Gustavo Villegas, Claudia Muñoz, Laura Serna, Sergio Alvarez, Hillary Protas, Ji Luo, Javad Sohankar, Yinghua Chen, Valentina Ghisays, Michael Malek-Ahmadi, Nicholas J. Ashton, Marisa N. Denkinger, Yuqi Cai, Yi Ran Xu, Beth Ostaszewski, Dennis J. Selkoe, Robert C. Alexander, Yakeel T. Quiroz, Yi Su, Kewei Chen, Rachelle S. Doody, Jessica B. Langbaum, Pierre N. Tariot, Kaycee M. Sink, Eric M. Reiman

**Affiliations:** a Genentech, Inc., South San Francisco, CA, USA; b F. Hoffmann-La Roche Ltd, Basel, Switzerland; c Neuroscience Group of Antioquia, University of Antioquia, Medellín, Colombia; d Roche Diagnostics, Penzberg, Germany; e Hospital San Juan de Dios de Yarumal, Antioquia, Colombia; f Hospital Pablo Tobón Uribe, Medellín, Colombia; g Banner Alzheimer’s Institute, Phoenix, AZ, USA; h Banner Sun Health Research Institute, Sun City, AZ, USA; i Department of Psychiatry and Neurochemistry, Institute of Neuroscience & Physiology, Sahlgrenska Academy at the University of Gothenburg, Mölndal, Sweden; j Ann Romney Center for Neurologic Diseases, Department of Neurology, Brigham and Women’s Hospital, Harvard Medical School, Boston, MA, USA; k Departments of Psychiatry and Neurology, Harvard Medical School, Massachusetts General Hospital, Charlestown, MA, USA

**Keywords:** Autosomal-dominant Alzheimer’s disease, Crenezumab, Amyloid, Prevention, PET, MRI, CSF, Blood biomarkers

## Abstract

**Background:**

The Alzheimer’s Prevention Initiative Autosomal Dominant Alzheimer’s Disease (ADAD) Colombia Trial evaluated the biological, cognitive, and clinical effects of crenezumab, an anti-oligomeric and monomeric amyloid-beta (Aβ) monoclonal antibody, in 30–60-year-old *PSEN1 E280A* mutation carriers without cognitive impairment from the world’s largest ADAD kindred, finding no significant treatment effects on Alzheimer’s disease progression. This article describes baseline biomarker, cognitive, and clinical measurements and placebo-related longitudinal changes in the randomised prevention trial’s mutation carrier and non-carrier groups.

**Methods:**

Crenezumab and placebo-treated mutation carriers and placebo-treated non-carriers were assessed using amyloid and fluorodeoxyglucose positron emission tomography (PET), magnetic resonance imaging, plasma, and optional tau PET and cerebrospinal fluid (CSF) biomarker, cognitive, and clinical measurements over 5–8 years.

**Findings:**

94% of the 252 kindred members (85 crenezumab-treated mutation carriers, 84 placebo-treated carriers, and 83 placebo-treated non-carriers) completed the trial. 55% of the carriers had baseline PET evidence of substantial Aβ plaques. 32.9% and 6.8% of amyloid PET-positive and PET-negative carriers, respectively, 36.5%, 13.2%, and 0% of pTau217-positive, pTau217-intermediate, and pTau217-negative carriers, respectively, and no non-carriers became cognitively impaired over the next 5 years. Carriers were distinguished from non-carriers by several baseline and longitudinal Aβ, tau, neurodegenerative, and inflammatory biomarker measures, but not by CSF oligomeric Aβ measurements.

**Interpretation:**

Despite the absence of significant treatment effects, these findings and the trial itself continue to inform the course of preclinical ADAD, advance Alzheimer’s disease prevention research, and provide a shared resource of data and samples for the field (ClinicalTrials.gov ID: NCT01998841; trial completed).

**Funding:**

National Institute on Aging, Banner Alzheimer’s Institute, Genentech, Inc., and F. Hoffmann-La Roche Ltd.

**Research in context**

**Evidence before this study**

A related article compares the effects of crenezumab, an anti-oligomeric and monomeric amyloid-beta (Aβ) monoclonal antibody, with placebo in cognitively unimpaired *PSEN1 E280A* mutation carriers from the 5–8-year Alzheimer’s Prevention Initiative Autosomal Dominant Alzheimer’s Disease (API ADAD) Colombia Trial. This article compares baseline and placebo-related biomarker, cognitive, and clinical findings in the trial’s mutation carriers and non-carriers. The trial and findings reported here were reviewed in context of available data related to the preclinical course of Alzheimer’s disease (AD) and advancement of AD research.

**Added value of this study**

The API ADAD Colombia Trial was a National Institutes of Health-funded trial of a putative AD-modifying treatment in cognitively unimpaired participants at biological risk for AD and was conducted in the world’s largest ADAD kindred. It introduced new scientific and resource-sharing paradigms for the accelerated evaluation and potential approval of AD prevention therapies. This report capitalised on comprehensive positron emission tomography, magnetic resonance imaging, cerebrospinal fluid, and plasma biomarker assessments to detect and track changes in Aβ, tau, neurodegenerative, inflammatory, and other biomarkers, inform subsequent clinical progression, and aid the design of future prevention trials. The findings support, complement, and further inform those generated in other ADAD and late-onset AD cohorts.

**Implications of all the available evidence**

Although crenezumab was not associated with significant pathophysiological or clinical effects in cognitively unimpaired mutation carriers when compared with placebo, the trial provided new information about biomarker trajectories and clinical progression in cognitively unimpaired ADAD mutation carriers, sample size estimates for secondary and primary ADAD prevention trials, and learnings for other AD prevention trials. The biomarker and cognitive findings and resource of data and samples described in this report continue to inform the course of preclinical ADAD and the evaluation of prevention therapies in cognitively unimpaired persons at genetic and/or biomarker risk for AD.

## Introduction

Alzheimer’s disease (AD) is the most common cause of age-related cognitive impairment in older people. In autosomal-dominant AD (ADAD), a presenilin 1 (*PSEN1*), presenilin 2 (*PSEN2*), or amyloid precursor peptide (*APP*) mutation causes an absolute or relative over-production in the amyloid-beta (Aβ) 42 peptide, leading mutation carriers to develop the biological and ensuing clinical features of AD at younger ages. In collaboration with the Alzheimer’s Prevention Initiative (API), University of Antioquia researchers identified and enrolled nearly 6000 members of the world’s largest known ADAD kindred in Antioquia, Colombia, into the API Colombia Registry,^1^ including about 1200 *PSEN1 E280A* mutation carriers who develop cognitive impairment due to AD at the median age of 44.^2^

While Aβ plaque-clearing antibodies have been shown to slow clinical decline in cognitively impaired patients,^3^^,^^4^ these and other disease-modifying treatments might have more benefit if introduced before the onset of cognitive impairment when the underlying disease is already extensive.^5^ In 2010, API introduced strategies to accelerate the evaluation of investigational AD-modifying drugs in people with no cognitive impairment at biological risk for AD, starting with a trial in members of the Colombian *PSEN1 E280A* kindred within 15 years of their estimated median age at mild cognitive impairment (MCI) onset, and vetted its ideas with numerous academic, industry, National Institutes of Health (NIH), U.S. Food and Drug Administration (FDA), European Medicines Agency, patient advocacy, and other stakeholders.

The Banner Alzheimer’s Institute secured an NIH grant (announced during the unveiling of the National Plan to Address AD in 2012), raised matching philanthropic funds, met with makers of promising amyloid-modifying drug therapies to encourage them to contribute their drug, expertise, co-leadership efforts, and funding to the trial, and establish a precedent-setting commitment to share trial data and samples after the trial was over.^6^^,^^7^ When Colombian ADAD kindred members were asked for their input, they expressed a preference for an investigational AD-modifying treatment that adequately reached its target in the brain and had preliminary information about its potential risks.

The decision was made to evaluate crenezumab, a humanised anti-Aβ monoclonal immunoglobulin G4 antibody that binds to Aβ monomers, oligomers, and fibrils, with the highest affinity for oligomers.^8^^,^^9^ The low effector function of its immunoglobulin G4 backbone and minimal binding to vascular amyloid deposits were thought to reduce the likelihood of brain vasculature inflammation and risk of amyloid-related imaging abnormalities,^8^ permitting administration of sufficiently high antibody doses to engage their target in the brain while maintaining an acceptable safety profile in a preclinical population.

The API ADAD trial and its related goals, methodology, baseline findings and longitudinal changes in the crenezumab and placebo-treated mutation carrier and non-carrier groups, have been described in separate reports.^10^^,^^11^ Participants in the randomised, double-blind, placebo-controlled trial were followed for 5–8 years until the last participant’s 60-month follow-up visit. Mutation carriers were randomised to crenezumab or placebo treatments to characterise the treatment’s clinical efficacy, biomarker effects, safety, and tolerability. Placebo-treated non-carriers from the kindred were included to avoid disclosure of the kindred members’ ADAD mutation status and advance the study of preclinical AD using baseline and placebo-related longitudinal data in the mutation carrier and non-carrier groups.^10^^,^^11^

All participants had serial amyloid positron emission tomography (PET), fluorodeoxyglucose (FDG) PET, volumetric magnetic resonance imaging (MRI), and plasma measurements of Aβ (A), tau (T), neurodegeneration (N), inflammation (I), and other pathophysiological changes. Approximately half had at least one cerebrospinal fluid (CSF) measurement of A/T/N/I/O and α-synuclein pathophysiology, including a recently developed measure of small oligomeric amyloid-beta (oAβ).^12^ About half of the participants had serial Tau PET scans after the technology became available in 2019. While Roche’s fully automated, first-generation assay was used to characterise plasma phosphorylated tau at threonine 217 (pTau217), its improved assay was performed *post hoc* to inform its relevance to the study of preclinical ADAD.

The effects of crenezumab vs. placebo in the mutation carriers are described elsewhere.^10^ Crenezumab was not associated with significant effects on API preclinical ADAD composite test score or the Free and Cued Selective Reminding Test-Cueing Index changes (the trial’s dual primary endpoints), time to MCI or dementia due to AD, or A/T/N/I/O biomarkers of disease progression. In *post hoc* and exploratory CSF biomarker comparisons, crenezumab was distinguished from placebo by increases in a new measure of small oAβ, total Aβ40, and attenuated declines in total Aβ42, providing suggestive but not definitive evidence that it engages and reduces CSF clearance of these oligomeric and monomeric Aβ species.

The objectives of the analyses in this report were to describe baseline findings, associations with age and placebo-related longitudinal A/T/N/I/O biomarker, cognitive and clinical measures in ADAD mutation carriers and non-carriers without cognitive impairment, and the likelihood of progressing to MCI or dementia in mutation carriers and non-carriers with and without amyloid PET or plasma pTau217 evidence of preclinical AD. This report provides new information about the performance of recently developed CSF oAβ and next-generation plasma pTau217 immunoassays in preclinical ADAD and considers potential implications of these findings and the trial’s historical context in preclinical detection, tracking, and prevention of AD.

## Methods

### Study design and participants

The API ADAD Colombia Trial, a randomised, double-blind, placebo-controlled prevention trial, which included a common-close design, was conducted at a primary site in Medellín, Colombia, according to the principles of the Declaration of Helsinki and Good Clinical Practice guidelines. The study methodology has been published previously.^10^^,^^11^ Three satellite sites in remote areas provided investigational product administration and safety assessments. The first participant was enrolled on 20 December, 2013 and the study was completed on 8 August, 2023.

### Randomisation and masking

Investigators and participants were blinded using an interactive voice- or web-based response system. Mutation carriers referred from the API Registry, screened, and determined to be eligible were randomly assigned to receive crenezumab or placebo and stratified by age, education, apolipoprotein E (*APOE*) ε4 carrier status, and baseline Clinical Dementia Rating (CDR) (zero vs. non-zero).^10^ Prospective trial participants who had low educational attainment but appeared eligible otherwise could receive a CDR score of 0.5 without having clinical evidence of a neurodegenerative disease and be classified as having no cognitive impairment because they did not meet the diagnostic criteria for MCI or dementia. Non-carriers followed an identical referral/screening process and were assigned to placebo for the previously noted reasons. Excluding the Independent Data Monitoring Committee and personnel preparing the study drug, all study personnel and participants were blinded to participants’ genetic status and treatment arm. Table 1 describes the schedule of assessments and methods for all biomarkers used in the study.

**Table 1:** Overview of biomarker methods and schedule of assessments.

	Methodology	Schedule of assessments
Amyloid PET	Florbetapir in SUVR and CL mean cortical-to-cerebellar SUVRs for baseline comparisons; mean cortical-white matter SUVRs for longitudinal comparisons	BL, 24 months, 60 months, and end of study
Tau PET	GTP1 entorhinal cortex SUVRs	Optional, 32–52 months, 45–67 months, and end of study
FDG PET	SUVRs (CMRgl decline) in an AD-related statistical ROI	BL, 3, 24, and 60 months, as well as end of study
vMRI	Whole brain Bilateral hippocampus Brain lateral ventricles	BL, 3, 6, 9, and 12 months, as well as every 6 months, and end of study
Plasma	Roche Elecsys® Total Aβ40, total Aβ42, pTau181, pTau217, & NfL (A/T/N); GFAP, sTREM2, YKL 40 (neuroinflammation)	BL, annual, and end of study
CSF	Roche Elecsys® Total Aβ40, total Aβ42, pTau181, tTau, NfL, & neurogranin (A/T/N); GFAP, sTREM2, YKL 40, IL-6, S100B (neuroinflammation); α-syn Selkoe lab assay^12^ for oAβ	Optional, BL, 24 months, 60 months, and end of study

α-syn, α-synuclein; Aβ, amyloid-beta; AD, Alzheimer’s disease; A/T/N, amyloid/tau/neurodegeneration; BL, baseline; CL, Centiloids; CMRgl, cerebral metabolic rate for glucose; CSF, cerebrospinal fluid; FDG, fluorodeoxyglucose; GFAP, glial fibrillary acidic protein; GTP1, Genentech Tau Probe 1; IL-6, interleukin-6; MRI, volumetric magnetic resonance imaging; NfL, neurofilament light; oAβ, oligomeric amyloid-beta; PET, positron emission tomography; pTau181/217, phosphorylated tau at threonine 181/217; ROI, region of interest; S100B, calcium-binding protein B; sTREM2, soluble triggering receptor expressed on myeloid cells 2; SUVR, standardised uptake value ratio; tTau, total tau; vMRI, volumetric magnetic resonance imaging; YKL 40, chitinase-3-like protein 1.

### Procedures

#### CSF and plasma biomarker collection and analysis

Participants were encouraged but not required to have lumbar punctures to collect CSF at baseline and Years 2 and 5 follow-up visits. Blood samples, including plasma, were collected from all participants at baseline and annual follow-up visits.

CSF biomarkers included those associated with Aβ pathophysiology (oAβ, total Aβ40 and total Aβ42), tau pathology (phosphorylated tau at threonine 181 [pTau181] and total tau [tTau]), neuronal degeneration (neurofilament light [NfL] and calcium-binding protein B [S100B]), and synaptic dysfunction (neurogranin); microglial activation (soluble triggering receptor expressed on myeloid cells 2 [sTREM2]), astroglial activation (glial fibrillary acidic protein [GFAP] and chitinase-3-like protein 1 [YKL 40]), and other inflammatory processes (interleukin-6 [IL-6]); and α-synuclein (α-syn) pathology. Plasma biomarkers included those associated with Aβ plaque deposition, Aβ-mediated tau pathophysiology and tau tangle deposition (pTau181, pTau217), neuronal injury and degeneration (NfL), and neuroinflammation (GFAP, sTREM2, and YKL 40).

CSF total Aβ42, tTau, and pTau181 were measured with Roche Diagnostics Elecsys® *in vitro* diagnostic medical device immunoassays and performed as described previously.^13^^,^^14^ CSF biomarkers, including total Aβ40, NfL, S100B, neurogranin, sTREM2, GFAP, YKL 40, α-syn, and IL-6, were measured, as were plasma biomarkers, including total Aβ40, total Aβ42, pTau181, first-generation pTau217, NfL, GFAP, sTREM2, and YKL 40, using Elecsys® NeuroToolKit robust prototype assays, as described previously.^15^

A CSF oAβ assay was performed *post hoc*. It used an Aβ-oligomer-preferring immunoassay on the SMCxPRO® (EMD Millipore) platform and is based on single-molecule-counting technology that allows a 20- to 100-fold increase in sensitivity compared with traditional immunodetection systems as previously described.^12^

A second-generation plasma pTau217 assay, run on Roche’s fully automated Elecsys® immunoassay platform, was performed *post hoc* and used for the main pTau217 analyses in this report. The assay, which uses a different pTau217 capture antibody and an N-terminal domain tau detector antibody, was recently shown to have a 0.94 (95% confidence interval [CI] 0.92, 0.96) receiver operating characteristic area under the curve (AUC) for the presence or absence of at least moderately frequent neuritic Aβ plaques using PET in older adults without cognitive impairment and without known ADAD mutations.^16^

#### PET image acquisition and analysis

PET images were acquired on a Siemens/CTI Biograph™ mCT and reconstructed using a standard iterative algorithm corrected for radiation attenuation and scatter. Florbetapir PET images were performed with a 10-mCi intravenous (IV) bolus florbetapir injection, 50-min radiotracer uptake period, and 20-min dynamic emission scan (4 × 5-min frames). For baseline assessments, mean cortical florbetapir standardised uptake value ratios (SUVRs) were calculated using inferior medial frontal gyrus, superior parietal cortex, lateral temporal cortex, posterior/anterior cingulate cortex, and precuneus regions of interest (ROIs) and a whole cerebellar reference ROI. Baseline mean cortical florbetapir SUVRs were transformed into Centiloids (CL), a standardised measurement used to characterise and compare PET measurements of neuritic plaque burden using different amyloid PET ligands, as previously described.^17^^,^^18^

**Fig. 1:**
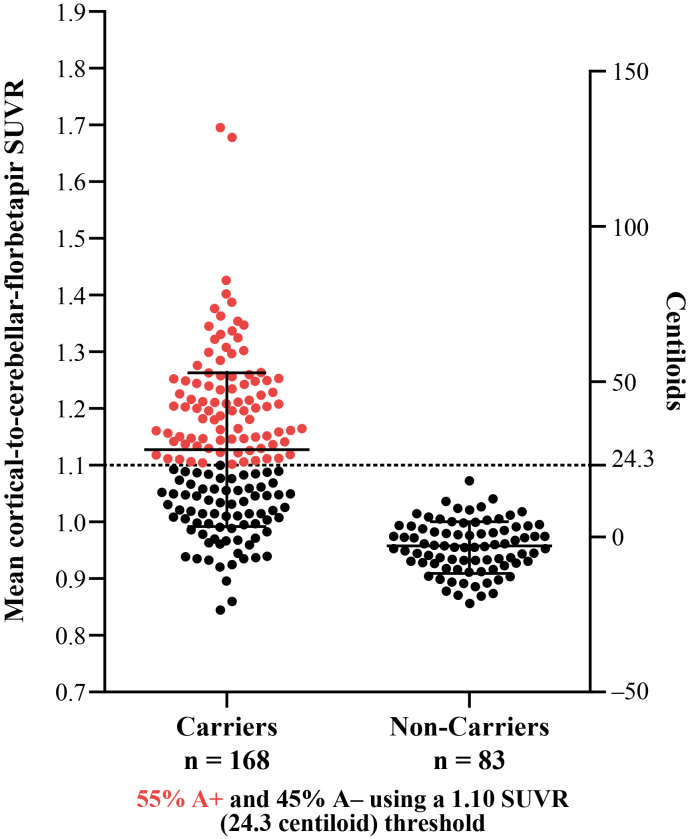
Baseline amyloid PET measurements in mutation carriers and non-carriers. Mean cortical florbetapir SUVR measurements of neuritic Aβ plaque burden and their corresponding CL were generated from each person’s florbetapir PET images as previously described.^14^^,^^15^ Horizontal dashed line indicates the threshold by which A+ scans were defined, based on ≥1.10, corresponding to ≥24.3 CL, based on the threshold used to distinguish between people with or without neuropathological evidence of at least moderately frequent neuritic Aβ plaques in a previous antemortem–postmortem comparison. A+, amyloid-positive; A–, amyloid-negative; Aβ, amyloid-beta; CL, Centiloids; PET, positron emission tomography; SUVR, standardised uptake value ratio.

Amyloid PET positivity was defined as SUVR ≥ 1.10 (24.3 CL), corresponding to evidence of at least moderately frequent neuritic Aβ plaques in an antemortem–postmortem neuropathological validation study.^19^ The presence of any measurable neuritic plaques was defined *post hoc* as SUVR >1.07 based on the maximum value of the entire young-adult and middle-aged mutation non-carrier group (Fig. 1). For longitudinal assessments, mean cortical SUVR changes were calculated using the same cerebral ROI, but a subcortical white matter (corpus callosum and centrum semiovale) reference ROI was used based on its established ability to detect longitudinal changes with improved precision and statistical power.^20^^,^^21^ Participants were scanned at baseline, Year 2, Year 5, and study completion (if more than 6 months had elapsed since the previous florbetapir PET scan).

FDG PET scans were performed in the resting state after at least a 4-h fast using a 5-mCi IV bolus FDG injection, 30-min radiotracer uptake period, and 30-min dynamic emission scan (6 × 5-min frames). SUVRs in an empirically predefined statistical ROI were calculated using bilateral posterior cingulate, medial and lateral parietal, frontal and temporal regions that decline preferentially, and white matter, cerebellum and somatosensory cortex regions that are preferentially spared in the early clinical stages of AD.^22^ Participants were scanned at baseline, Week 12, Year 2, Year 5, and study completion (only if more than 6 months had elapsed since the previous FDG PET scan).

Genentech Tau Probe 1 (GTP1) PET images of tau tangle burden were performed using 7-mCi IV bolus GTP1 injection, 60-min radiotracer uptake period, and 30-min dynamic emission scan (6 × 5-min frames). Mean SUVR values were calculated for the entorhinal cortex using an inferior cerebellum reference region, defined based on the joint analysis of a cerebellum atlas in Montreal Neurological Institute space and the FreeSurfer-defined cerebellar cortex.^23^ Unlike the florbetapir PET and FDG PET scans required as part of the main study, tau PET scans were part of an optional sub-study initiated when GTP1 was approved for use by local health authorities and after the start of the main study, preventing the acquisition of pre-treatment baseline scans. First scans were acquired as soon as eligible people consented to participate in the sub-study, therefore the timing of the scans relative to each participant’s start of treatment was variable, ranging from Week 130 to Week 224, depending on date of entrance into the main study. Second scans were acquired as close as possible to Year 5 (as late as possible in the Week 248–260 window) regardless of the timing of the first scan so that, although the relative separations between the first and second scans varied, all participants would have approximately the same treatment duration. A third optional scan was acquired at study completion if more than 6 months had elapsed since the previous GTP1 PET scan.

All PET analyses were performed using a hybrid pipeline combining the PET Unified Pipeline^24^^,^^25^ and Statistical Parametric Mapping (SPM; https://www.fil.ion.ucl.ac.uk/spm). Image frames were corrected for motion, summed, and co-registered with baseline MRIs, and transformed into Montreal Neurological Institute space using SPM. Mean SUVRs were computed in either native or template space, depending on the radiotracer method.

#### MRI acquisition and analyses

MRI exams were performed on a 3T Siemens Tim Trio at Hospital Pablo Tobón Uribe and consisted of several sequences for safety, structural, and functional assessments. For volumetric measurements, a 3D T1 MP-RAGE was used (repetition time 1.8 s, echo time 2.5 ms, inversion time 900 ms, field of view 24 cm, 1.25 × 1.25 mm^2^ in-plane resolution, 1.2-mm slices). MRIs were acquired at baseline, Weeks 12, 24, 36, and 52, and then every 6 months until study completion. In participants who were switched from subcutaneous to IV treatment, an additional MRI was performed 3 months after the first IV dose and every 6 months thereafter.

Bilateral hippocampal and brain lateral ventricle volumes at each visit were calculated using FreeSurfer^26^ to compute longitudinal percentage changes from baseline at each follow-up visit. Baseline whole brain volumes were measured using the same FreeSurfer methods, while longitudinal percentage changes were measured using an iterative principal component analysis (IPCA) applied to pairs of baseline and follow-up images.^27^ For each participant, the inherent bias in the IPCA follow-up-to-baseline whole brain volume changes was corrected by incorporating the average of 12-week-to-baseline and baseline-to-12-week whole brain volume changes.

### Outcomes

Pre-specified secondary biomarker outcomes were baseline and annualised rates of change in amyloid PET, tau PET, FDG PET scans; volumetric MRI of whole brain, bilateral hippocampus, and brain lateral ventricles; and CSF pTau181, tTau, and NfL. Annual changes in FDG PET were assessed using an empirically predefined statistical ROI preferentially affected by cerebral metabolic glucose rate decline in AD.^22^ An entorhinal-to-inferior cerebellum SUVR was used as the tau burden index.^23^^,^^24^ Exploratory biomarker outcomes were baseline values of, and annualised rate of change in, CSF and plasma biomarkers that assess amyloid pathology (total Aβ40, total Aβ42, oAβ), tau pathology (tTau, pTau), neurodegeneration (NfL, S100B), synaptic dysfunction (neurogranin), microglial (sTREM2) and astroglial activation (GFAP, YKL 40), other neuropathologies (α-syn), and inflammation (IL-6).^12^

### Ethics

Trial and recruitment materials were approved by the ethics committee at the Hospital Pablo Tobón Uribe and the Colombian Health Authority, Instituto Nacional de Vigilancia de Medicamentos y Alimentos (ethics approval number PI-BA-796), and monitored by an Independent Data and Safety Monitoring committee. All participants and study partners provided written informed consent.

### Statistics

Longitudinal biomarker trajectories were assessed descriptively to characterise the natural history of the cohort. Unadjusted mean changes from baseline and corresponding 95% confidence intervals (CIs) were calculated at each visit for placebo-treated mutation carriers (stratified by baseline amyloid PET status) and non-carriers. Group differences over time were evaluated visually; non-overlapping 95% CIs between groups were interpreted as a descriptive indicator of nominally significant divergence. Formal statistical hypothesis testing was not performed for these specific longitudinal comparisons. To visualise the longitudinal trajectory of tau PET SUVR across groups despite inconsistent follow-up intervals, we employed LOESS (Locally Estimated Scatterplot Smoothing) for each group. This non-parametric approach allowed for a semi-quantitative characterisation of temporal trends by pooling local data points to estimate the group-level mean trajectory over time. Additional exploratory plots include a box plot of CL values at baseline in mutation carriers compared with non-carriers, as well as plots showing the relationship between age and CL values and second-generation plasma pTau217 at baseline (in mutation carrier and non-carrier groups separately). Kaplan–Meier survival curves showing the cumulative incidence (probability) of conversion to MCI or AD split by biomarker levels within mutation carriers and separately compared with the combined mutation non-carrier population were used to characterise the prognostic value of different biomarker strategies for identifying patient populations enriched for those likely to progress to MCI or AD. All analyses examine the intent-to-treat (ITT) population of the trial—all randomised participants, whether or not the participant received the assigned treatment. Only participants in the ITT with available measurements at baseline were included—missing post-baseline measurements were not included in any of the longitudinal assessments. All available data (only post-baseline data) from the tau PET analysis were included.

The trial’s longitudinal results were used to estimate: 1) the number of Aβ PET-positive mutation carriers without cognitive impairment needed to detect a 50% reduction in MCI or dementia progression to MCI or dementia in a 60-month, placebo-controlled, secondary prevention trial; 2) the number of amyloid PET-negative mutation carriers needed to detect a 50% reduction in MCI or dementia progression in a 60-month primary prevention trial; 3) the number of Aβ-negative mutation carriers needed to detect a 75% reduction in progression to a positive amyloid PET scan in a 60-month primary prevention trial with 80% power and two-tailed 0.05 alpha (see Supplementary Methods for further information). In all cases, the sample size estimates refer to the number of participants who complete the trial. For exploratory estimation of prevention trial sample size based on amyloid PET progression, longitudinal participant-level amyloid PET measurements in baseline amyloid-negative mutation carriers were analysed using linear mixed-effects models to estimate individual trajectories and predicted time to amyloid positivity threshold crossing over a 60-month period. Statistical analyses and sample size estimations were performed using **R version 4.4.3.** This trial was registered with ClinicalTrials.gov on 22 November 2013, number NCT01998841 (https://www.clinicaltrials.gov/study/NCT01998841).

### Role of funders

The API ADAD Colombia Trial was a public–private partnership involving Banner Alzheimer’s Institute, the Neuroscience Group of Antioquia, Genentech, Inc., and its parent organisation F. Hoffmann-La Roche, and NIH. It was sponsored by the National Institute on Aging (NIA), Banner Alzheimer’s Institute, Genentech, Inc., and F. Hoffmann-La Roche Ltd, and jointly managed by Genentech, Inc., F. Hoffmann-La Roche Ltd, and Banner Alzheimer’s Institute. Banner Alzheimer’s Institute, Genentech, Inc., and F. Hoffmann-La Roche Ltd shared responsibility for study design, study implementation, data collection, data management, data analysis, data interpretation, and preparation, review, approval, and decision to submit the manuscript for publication. The NIA served in an advisory capacity and approved data monitoring committee appointments.

The authors were not paid to write this article by a pharmaceutical company or other agency. Authors were not precluded from accessing data in the study and accept responsibility to submit for publication.

## Results

### Baseline characteristics

**Fig. 2:**
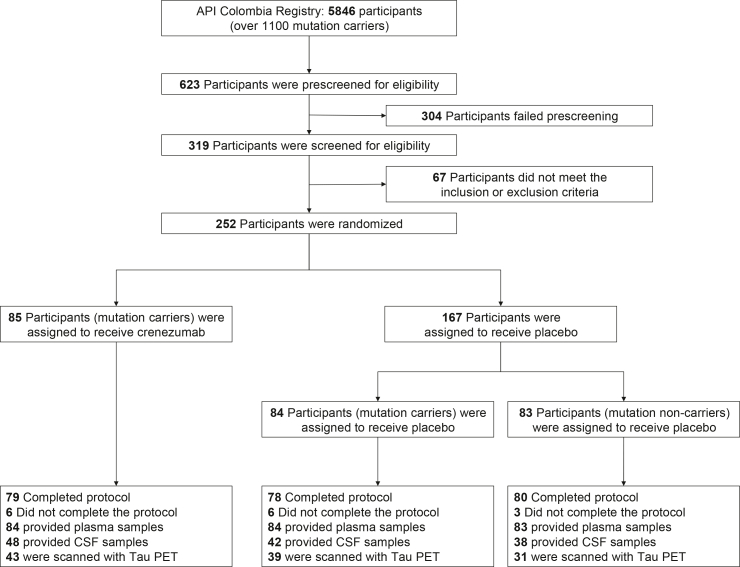
Trial profile including biomarker assessments. API, Alzheimer’s Prevention Initiative; PET, positron emission tomography.

Fig. 2 shows the number of pre-screened, screened, enrolled, and biomarker-assessed research participants.^1^ The Colombian API Registry enrolled 5846 *PSEN1 E280A* kindred members, including nearly 1200 mutation carriers. 252 participated in the trial, including 179 *PSEN1 E280A* mutation carriers (85 randomised to crenezumab and 84 to placebo) and 83 placebo-treated non-carriers. They were enrolled between December 2013 and February 2017, and their data were collected through March 2022.

Table 2 shows baseline demographic, *APOE* ε4, clinical, cognitive, and PET, MRI, CSF, and plasma biomarker characteristics in the *PSEN1 E280A* mutation carrier and non-carrier groups. The mutation carriers’ mean age was 36.8 years, 7.2 years before their estimated median age of 44 at MCI onset. The mutation carriers were significantly younger than non-carriers (mean age 43.3 years), at least partly due to the paucity of mutation carriers without cognitive impairment in their mid-to-late 50s. After adjusting baseline measurements for age, the aggregate mutation carrier group was distinguished from the non-carrier group by small but nominally significant elevations in baseline Functional Assessment Staging Tool for Dementia, CDR—Sum of Boxes (CDR-SB), and percentage of CDR >0 global scores and by small but nominally significant lower mean MMSE, API ADAD Composite, Free and Cued Selective Reminding Test Scores.

**Table 2:** Baseline demographics and characteristics.

	Crenezumab—carrier (n = 85)	Placebo—carrier (n = 84)	Placebo—non-carrier (n = 83)	Effect size and nominal P-value (all carriers vs. non-carriers, adjusted for age)^a^	Effect size and nominal P-value (placebo carriers vs. non-carriers, adjusted for age)^a^
Mean age, years (SD)	36.8 (5.3)	36.9 (6.3)	43.3 (7.2)	W = 3410.5, P < 0.001, r_rb = −0.51^b^	W = 1718.5, P < 0.001, r_rb = −0.51^b^
Female, n (%)	44 (51.8)	59 (70.2)	57 (68.7)	OR = 0.66 (95% CI: 0.35–1.22), P = 0.189	OR = 1.14 (95% CI: 0.55–2.39), P = 0.717
Years of education < 9, n (%)^c^	37 (43.5)	39 (46.4)	45 (54.2)	OR = 1.22 (95% CI: 0.67–2.26), P = 0.521	OR = 1.35 (95% CI: 0.67–2.78), P = 0.413
*APOE* ε4 status, n (%)^c^					
Carrier	19 (22.4)	17 (20.2)	19 (22.9)	OR = 0.89 (95% CI: 0.45–1.82), P = 0.749	OR = 0.84 (95% CI: 0.37–1.91), P = 0.684
Non-carrier	66 (77.6)	67 (79.8)	64 (77.1)		
Mean CDR-GS non-zero (%)^c^	8 (9.4)	10 (11.9)	5 (6.0)	OR = 3.40 (95% CI: 1.13–12.10), P = 0.040	OR = 3.15 (95% CI: 0.94–12.05), P = 0.073
Mean CDR-SB (SD)	0.16 (0.38)	0.14 (0.43)	0.05 (0.17)	F(1, 249) = 10.52, P = 0.001, partial eta squared = 0.041	F(1, 164) = 4.74, P = 0.031, partial eta squared = 0.028
Mean API ADAD composite (SD)	81.935 (8.824)	80.444 (11.291)	83.696 (9.829)	F(1, 247) = 27.23, P < 0.001, partial eta squared = 0.099	F(1, 164) = 23.6, P < 0.001, partial eta squared = 0.126
Mean FCSRT-CI (SD)	0.776 (0.165)	0.763 (0.199)	0.828 (0.140)	F(1, 247) = 18.59, P < 0.001, partial eta squared = 0.07	F(1, 163) = 10.06, P = 0.002, partial eta squared = 0.058
Mean MMSE total score (SD)	28.847 (1.268)	28.786 (1.506)	29.193 (1.030)	F(1, 249) = 14.08, P < 0.001, partial eta squared = 0.054	F(1, 164) = 11.85, P = 0.001, partial eta squared = 0.067
Mean NPI total score (SD)	0.262 (0.893)	0.643 (2.160)	0.370 (1.946)	F(1, 246) = 0.05, P = 0.825, partial eta squared = 0	F(1, 162) = 0.06, P = 0.804, partial eta squared = 0
Mean FAST (SD)	1.094 (0.294)	1.131 (0.373)	1.012 (0.110)	F(1, 249) = 16.07, P < 0.001, partial eta squared = 0.061	F(1, 164) = 13.77, P < 0.001, partial eta squared = 0.077
PET					
Amyloid PET-positive participants: SUVR ≥ 1.1 (24.3 CL), %	61.2	48.8	0	OR = 0.00 (95% CI: 0.00–0.01), P < 0.001^d^	OR = 0.00 (95% CI: 0.00–0.02), P < 0.001^d^
Mean amyloid PET, SUVR (SD)^e^	1.15 (0.15)	1.11 (0.12)	0.96 (0.04)	F(1, 249) = 267.06, P < 0.001, partial eta squared = 0.517	F(1, 164) = 173.47, P < 0.001, partial eta squared = 0.514
Mean amyloid PET, CL (SD)	33.11 (27.22)	25.36 (21.43)	−2.18 (8.23)	F(1, 249) = 267.06, P < 0.001, partial eta squared = 0.517	F(1, 164) = 173.47, P < 0.001, partial eta squared = 0.514
Mean FDG PET, SUVR (SD)	1.29 (0.05)	1.29 (0.04)	1.30 (0.04)	F(1, 249) = 21.86, P < 0.001, partial eta squared = 0.081	F(1, 164) = 17.65, P < 0.001, partial eta squared = 0.097
vMRI, mean (SD) Participants, n					
vMRI whole brain, μL	1187651.833 (116137.386); n = 84	1150080.083 (89912.350); n = 84	1144841.422 (112438.683); n = 83	F(1, 248) = 0.51, P = 0.475, partial eta squared = 0.002	F(1, 164) = 0.74, P = 0.392, partial eta squared = 0.004
vMRI bilateral hippocampus, μL	8374.927 (849.131); n = 85	8243.615 (726.938); n = 84	8241.700 (763.589); n = 83	F(1, 249) = 0.43, P = 0.511, partial eta squared = 0.002	F(1, 164) = 0.9, P = 0.344, partial eta squared = 0.005
vMRI brain lateral ventricles, μL	13386.786 (6119.661); n = 85	13387.295 (5641.237); n = 84	14138.036 (6746.178); n = 83	F(1, 249) = 1.42, P = 0.235, partial eta squared = 0.006	F(1, 164) = 0.69, P = 0.407, partial eta squared = 0.004
CSF, mean (SD) Participants, n					
Aβ42, pg/mL^f^	890.94 (328.74); n = 48	850.91 (317.84); n = 41	1402.61 (326.06); n = 37	F(1, 123) = 96.51, P < 0.001, partial eta squared = 0.44	F(1, 75) = 71.39, P < 0.001, partial eta squared = 0.488
Aβ40, pg/mL^f^	13628.13 (3714.62); n = 48	13616.19 (3608.81); n = 42	14799.19 (4281.55); n = 37	F(1, 124) = 0.78, P = 0.38, partial eta squared = 0.006	F(1, 76) = 0.81, P = 0.372, partial eta squared = 0.011
oAβ, pg/mL	3540.44 (1943.49); n = 42	3718.64 (2085.57); n = 38	3886.28 (1898.00); n = 34	F(1, 111) = 0.5, P = 0.48, partial eta squared = 0.005	F(1, 69) = 0.35, P = 0.554, partial eta squared = 0.005
pTau181, pg/mL^f^	20.43 (18.20); n = 48	19.32 (11.62); n = 42	11.44 (4.80); n = 37	F(1, 124) = 37.42, P < 0.001, partial eta squared = 0.232	F(1, 76) = 25.23, P < 0.001, partial eta squared = 0.249
tTau, pg/mL^f^	197.91 (123.94); n = 48	202.99 (93.44); n = 42	138.49 (57.30); n = 36	F(1, 123) = 22.19, P < 0.001, partial eta squared = 0.153	F(1, 75) = 17.14, P < 0.001, partial eta squared = 0.186
NfL (log_10_), pg/mL^f^	1.71 (0.33); n = 48	1.72 (0.20); n = 42	1.66 (0.15); n = 37	F(1, 124) = 12.51, P = 0.001, partial eta squared = 0.092	F(1, 76) = 11.66, P = 0.001, partial eta squared = 0.133
Neurogranin, pg/mL^f^	1051.19 (622.25); n = 48	1110.30 (658.52); n = 42	804.66 (350.03); n = 37	F(1, 124) = 4.87, P = 0.029, partial eta squared = 0.038	F(1, 76) = 2.76, P = 0.101, partial eta squared = 0.035
α-syn, pg/mL^f^	324.49 (247.35); n = 48	464.03 (690.28); n = 42	251.59 (152.13); n = 37	F(1, 124) = 7.8, P = 0.006, partial eta squared = 0.059	F(1, 76) = 6.71, P = 0.012, partial eta squared = 0.081
GFAP, pg/mL^f^	4674.17 (2842.91); n = 48	4762.38 (1946.85); n = 42	4073.24 (1979.91); n = 37	F(1, 124) = 12.12, P = 0.001, partial eta squared = 0.089	F(1, 76) = 11.94, P = 0.001, partial eta squared = 0.136
sTREM2, ng/mL^f^	7.52 (2.65); n = 48	7.67 (2.19); n = 42	7.44 (2.11); n = 37	F(1, 124) = 2.16, P = 0.144, partial eta squared = 0.017	F(1, 76) = 1.35, P = 0.249, partial eta squared = 0.017
YKL 40, ng/L^f^	87882.71 (51123.54); n = 48	97458.81 (39429.08); n = 42	90774.32 (31519.41); n = 37	F(1, 124) = 3.72, P = 0.056, partial eta squared = 0.029	F(1, 76) = 4.68, P = 0.034, partial eta squared = 0.058
IL-6 (log_10_), ng/L^f^	0.57 (0.10); n = 48	0.56 (0.22); n = 42	0.55 (0.12); n = 37	F(1, 124) = 0.12, P = 0.728, partial eta squared = 0.001	F(1, 76) = 0.67, P = 0.417, partial eta squared = 0.009
S100B, μg/L^f^	0.70 (0.18); n = 48	0.77 (0.28); n = 42	0.68 (0.28); n = 37	F(1, 123) = 2.6, P = 0.109, partial eta squared = 0.021	F(1, 75) = 2.84, P = 0.096, partial eta squared = 0.036
Plasma, mean (SD), participants, n					
Aβ42, pg/mL^f^	27.40 (5.58); n = 84	25.93 (5.86); n = 84	24.78 (14.32); n = 82	F(1, 247) = 19.41, P < 0.001, partial eta squared = 0.073	F(1, 163) = 12.17, P = 0.001, partial eta squared = 0.069
Aβ40, pg/mL^f^	285.19 (52.46); n = 84	276.04 (68.42); n = 84	326.84 (189.40); n = 83	F(1, 248) = 10.48, P = 0.001, partial eta squared = 0.041	F(1, 164) = 7.98, P = 0.005, partial eta squared = 0.046
pTau181 (log_10_), pg/mL^f^	−0.01 (0.19); n = 84	−0.01 (0.22); n = 84	−0.25 (0.11); n = 83	F(1, 248) = 169.79, P < 0.001, partial eta squared = 0.406	F(1, 164) = 92.79, P < 0.001, partial eta squared = 0.361
First-generation pTau217 (Log_10_), pg/mL^f^	−0.60 (0.26); n = 84	−0.57 (0.30); n = 84	−0.85 (0.25); n = 83	F(1, 248) = 66.69, P < 0.001, partial eta squared = 0.212	F(1, 164) = 43.61, P < 0.001, partial eta squared = 0.21
Second-generation pTau217 (log_10_), pg/mL^f^	−0.462 (0.288); n = 79	−0.466 (0.331); n = 77	−0.918 (0.164); n = 79	F(1, 232) = 228.62, P < 0.001, partial eta squared = 0.496	F(1, 153) = 124.37, P < 0.001, partial eta squared = 0.448
NfL (log_10_), pg/mL^f^	0.09 (0.21); n = 84	0.13 (0.17); n = 84	0.05 (0.19); n = 83	F(1, 248) = 17.85, P < 0.001, partial eta squared = 0.067	F(1, 164) = 18.75, P < 0.001, partial eta squared = 0.103
GFAP (log_10_), pg/mL^f^	1.83 (0.30); n = 84	1.86 (0.33); n = 84	1.54 (0.23); n = 83	F(1, 248) = 132.32, P < 0.001, partial eta squared = 0.348	F(1, 164) = 95.74, P < 0.001, partial eta squared = 0.369
sTREM2 (log_10_), ng/mL^f^	0.66 (0.18); n = 84	0.63 (0.18); n = 84	0.69 (0.22); n = 83	F(1, 248) = 0.45, P = 0.502, partial eta squared = 0.002	F(1, 164) = 0.03, P = 0.856, partial eta squared = 0
YKL 40 (log_10_), μg/L^f^	1.47 (0.25); n = 84	1.43 (0.19); n = 84	1.54 (0.28); n = 83	F(1, 248) = 0.16, P = 0.689, partial eta squared = 0.001	F(1, 164) = 0.08, P = 0.781, partial eta squared = 0

Baseline data from the crenezumab-treated group have been published previously.^10^

α-syn, α-synuclein; Aβ, amyloid-beta; API ADAD composite, Alzheimer’s Prevention Initiative preclinical autosomal-dominant Alzheimer’s disease composite test score; *APOE*, apolipoprotein E; CDR-GS, Clinical Dementia Rating Scale—Global Score; CDR-SB, Clinical Dementia Rating Scale—Sum of Boxes; CL, Centiloids; CSF, cerebrospinal fluid; FAST, Functional Assessment Staging Test; FCSRT-CI, Free and Cued Selective Reminding Test-Cueing Index; FDG, fluorodeoxyglucose; GFAP, glial fibrillary acidic protein; GTP1, Genentech Tau Probe 1; IL-6, interleukin-6; MMSE, Mini-Mental State Examination; NfL, neurofilament light; NPI, Neuropsychiatric Inventory; oAβ, oligomeric amyloid-beta; PET, positron emission tomography; pTau181/217, phosphorylated tau at threonine 181/217; S100B, calcium-binding protein B; SD, standard deviation; sTREM2, soluble triggering receptor expressed on myeloid cells 2; SUVR, standardised uptake value ratio; tTau, total tau; vMRI, volumetric magnetic resonance imaging; YKL 40, chitinase-3-like protein 1.

a Effect sizes and P-values for categorical variables were estimated using logistic regression; Quade’s non-parametric ANCOVA was used for continuous variables.

b Effect sizes and P-values for differences in age by group were estimated using Mann–Whitney U and Rank–Biserial Correlation (r_rb).

c Stratification variables.

d Due to quasi-complete separation in the data, we utilised Firth’s penalised likelihood logistic regression to estimate the effect.

e Whole cerebellum used as the reference region; threshold >1.1 defined as positive. Baseline tau PET measurements are not available since GTP1 PET was introduced later in the trial.

f A subset of participants took part in the optional CSF sub-study.

127 participants (∼50%) provided CSF samples at one or more of their baseline, Year 2, and Year 5 visits. After adjusting baseline measurements for age, mutation carriers were distinguished from non-carriers by nominally significant lower CSF total Aβ42 (P < 0.001; F(1, 123) = 96.51; Quade’s non-parametric ANCOVA), plasma total Aβ40 (P < 0.001; F(1, 248) = 10.48), and FDG PET measurements in an AD-related statistical ROI (P < 0.001; F(1, 249) = 21.86); and higher amyloid PET (P < 0.001; F(1, 249) = 267.06), CSF pTau181 (P < 0.001; F(1, 124) = 37.42), tTau (P < 0.001; F(1, 123) = 22.19), NfL (P < 0.001; F(1, 124) = 12.51), neurogranin (P = 0.029; F(1, 124) = 4.87), GFAP (P < 0.001; F(1, 124) = 12.12), α-syn (P = 0.006; F(1, 124) = 7.8), plasma Aβ42 (P < 0.001; F(1, 247) = 19.41), pTau217 (P < 0.001; F(1, 232) = 228.62), pTau181 (P < 0.001; F(1, 248) = 169.79), NfL (P < 0.001; F(1, 248) = 17.85) and GFAP (P < 0.001; F(1, 248) = 132.32) measurements. There were no other nominally significant demographic, *APOE* ε4, or baseline cognitive, clinical, or biomarker differences in the aggregate carrier and non-carrier groups.

Fig. 1 shows baseline amyloid PET measurements in the mutation carrier and non-carrier groups. 93 (55%) of 168 carriers were amyloid PET-positive and 75 (45%) were amyloid PET-negative. At baseline, all non-carriers were amyloid PET-negative. Mean SUVR was 29.3 CL in carriers, suggesting a relatively early stage of preclinical AD, and −2.2 CL in non-carriers.

**Fig. 3:**
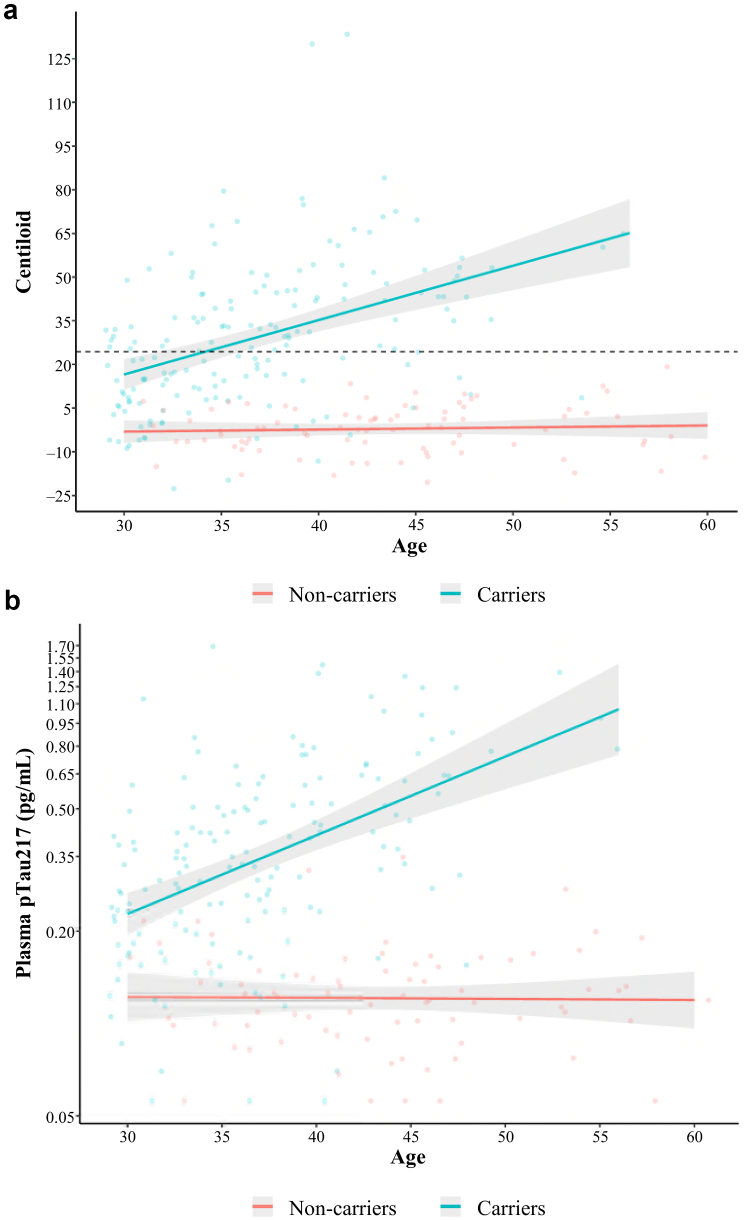
Associations between baseline (a) florbetapir PET (CL) and (b) plasma pTau217 biomarker measurements of Aβ plaque deposition and age. Least squares linear regression model fits and 95% CIs by group are shown. The dashed horizontal line in (a) shows the amyloid PET positivity cutoff (CL ≥ 24.3). Aβ, amyloid-beta; CI, confidence interval; CL, Centiloids; PET, positron emission tomography; pTau217, phosphorylated tau at threonine 217.

Fig. 3 shows nominally significant associations of baseline amyloid PET SUVR (adjusted R^2^ = 0.19; P = 2.31 × 10^−9^) and plasma pTau217 (adjusted R^2^ = 0.22; P = 2.61 × 10^−10^), with older age in the mutation carriers but not in the non-carriers.

### Performance of a fully automated pTau217 assay in ADAD

In a *post hoc* analysis, Roche’s second-generation pTau217 assay had a moderately high discriminative accuracy in distinguishing between *PSEN1 E280A* mutation carriers without cognitive impairment with a positive vs. negative amyloid PET scan (AUC 0.78 [95% CI: 0.70, 0.85]; Supplementary Fig. S1), but it was notably less accurate than the 0.94 AUC (95% CI: 0.92, 0.96) in older adults without cognitive impairment and without known ADAD mutations.^16^

### Prognostic value of amyloid PET and plasma pTau217 in ADAD

**Fig. 4:**
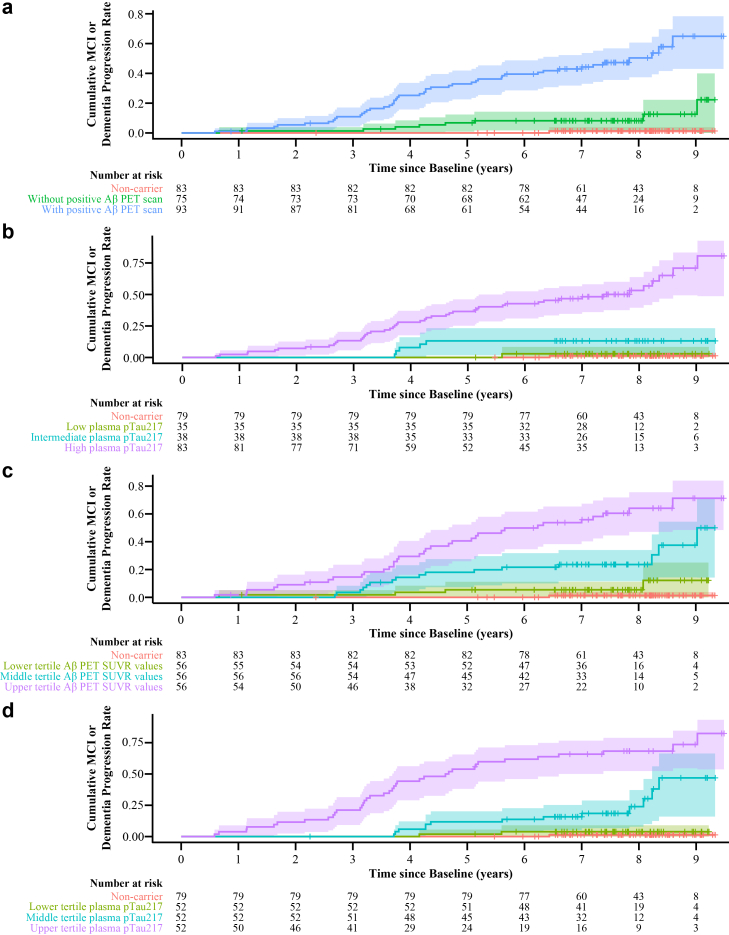
Cumulative rate of progression to MCI or AD diagnosis according to baseline biomarker measurements in mutation carriers (a) with or without a positive amyloid PET scan; (b) with high, intermediate, or low plasma pTau217 measurements; (c) by tertiles of CL; and (d) by tertiles of plasma pTau217. A positive amyloid PET scan is defined as SUVR ≥ 24.3 CL. High, intermediate, and low plasma pTau217 measurements were defined using the two-threshold method as ≥0.328 ng/mL, 0.201–0.328 ng/mL, and <0.201 ng/mL, respectively. Upper, middle, and lower tertiles for amyloid PET SUVR values were >39.0 CL, 16.5–39.0 CL, and <16.5 CL, respectively. Mutation non-carriers are included in all plots for comparison. Aβ, amyloid-beta; AD, Alzheimer’s disease; CL, Centiloids; MCI, mild cognitive impairment; PET, positron emission tomography; pTau217, phosphorylated tau at threonine 217; SUVR, standardised uptake value ratio.

Fig. 4 provides information about the ability of baseline amyloid PET radiotracer method and plasma pTau217 to predict subsequent rates of progression to MCI or dementia in mutation carriers without cognitive impairment over the next 5–9 years.

Data shown are for amyloid biomarker measurements divided into individuals with or without a positive amyloid PET scan using the 24.3 CL threshold (Fig. 4a), and high, intermediate, or low plasma pTau217 measurements using the two-threshold strategy described above (Fig. 4b). The Kaplan–Meier estimated cumulative probability of progressing to MCI or dementia over the next 5 years was 32.9% among the 93 amyloid PET-positive carriers and 6.8% among the 75 amyloid PET-negative carriers. Furthermore, the estimated probability of progression was 36.5% for the 83 pTau217-positive carriers, 13.2% for the 38 pTau217-intermediate carriers, and 0% for the 35 pTau217-negative carriers; no non-carriers progressed during this period.

Data are also shown when these biomarker measurements are divided into tertiles (Fig. 4c and d), the strategy used to assess subsequent rates of progression in older research participants without cognitive impairment from the A4 and LEARN studies, to compare the prognostic value of these AD biomarkers in ADAD and late-onset AD. When baseline PET and pTau217 data are divided into tertiles (e.g., when PET measurements are divided into those with values < 16.5 CL, 16.5–39.0 CL, and >39.0 CL, and pTau217 measurements are divided into those with <0.258 pg/mL, 0.258–0.476 pg/mL, and >0.476 pg/mL), the Kaplan–Meier estimated cumulative probability of progressing to MCI or dementia in 5 years was 40.6% for the 56 carriers in the highest PET tertile and 53.8% for the 52 carriers in the highest plasma pTau217 tertile. This is similar to the subsequent rates of clinical progression observed in older adults in the highest biomarker tertile but without known ADAD mutations. None of the mutation non-carriers progressed to MCI or dementia over 5 years.

### Longitudinal trajectories and statistical power estimates

**Fig. 5:**
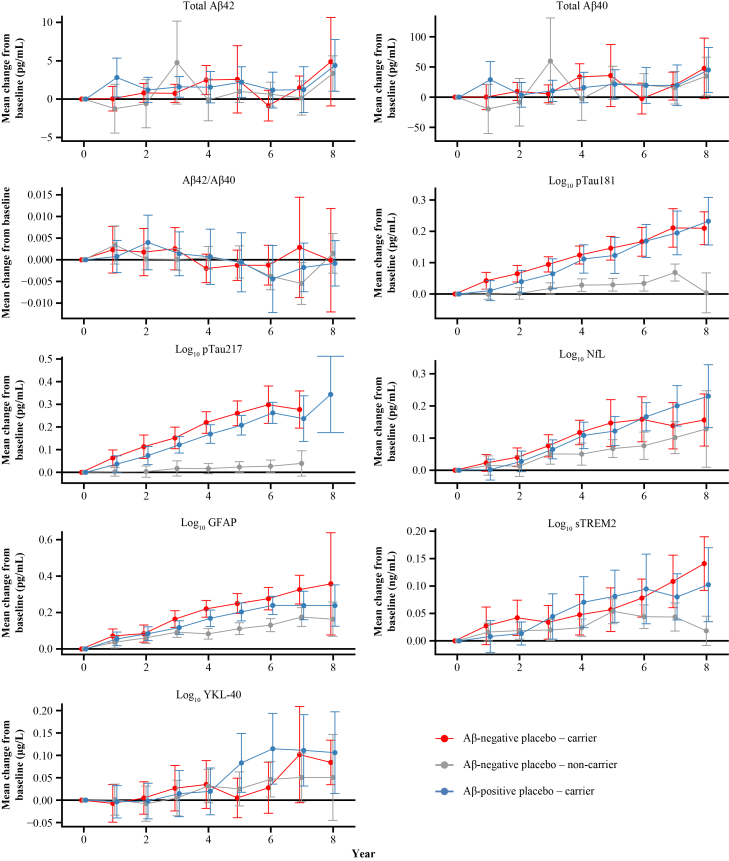
Longitudinal trajectories for plasma biomarker changes in placebo-treated mutation carriers with a positive or negative baseline amyloid PET scan and non-carriers, all of whom had a negative baseline amyloid PET scan. A positive amyloid PET scan is defined as ≥24.3 CL. Point estimates of unadjusted mean change from baseline with 95% CI are shown. Aβ, amyloid-beta; CI, confidence interval; GFAP, glial fibrillary acidic protein; NfL, neurofilament light; pTau181/217, phosphorylated tau at threonine 181/217; sTREM2, soluble triggering receptor expressed on myeloid cells 2; YKL-40, chitinase-3-like protein 1.

**Fig. 6:**
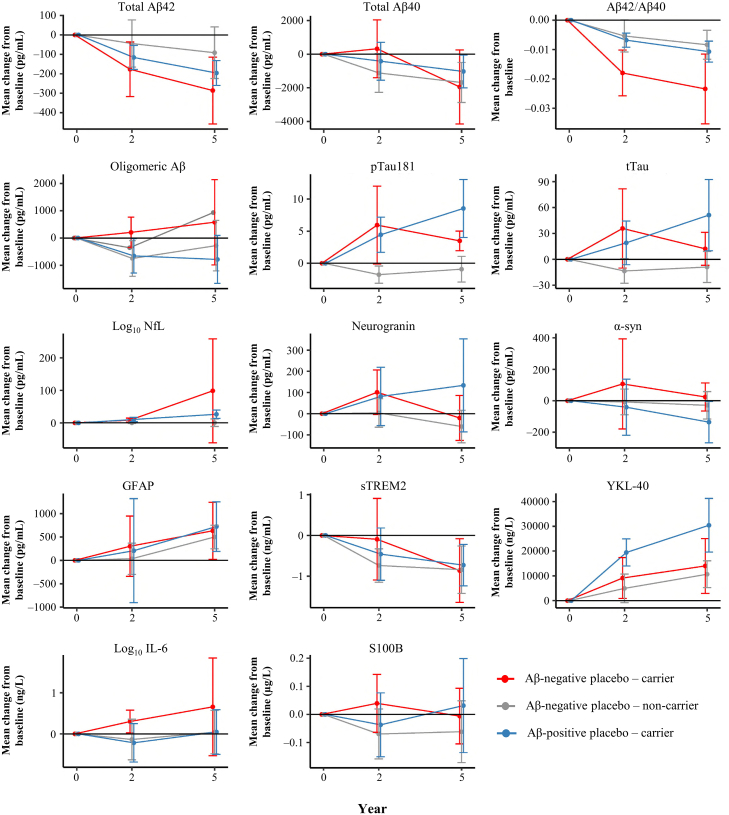
Longitudinal trajectories for CSF biomarker changes in placebo-treated mutation carriers with a positive or negative baseline amyloid PET scan and non-carriers, all of whom had a negative baseline amyloid PET scan. A positive amyloid PET scan is defined as ≥24.3 CL. Point estimates of unadjusted mean change from baseline with 95% CI are shown. Aβ, amyloid-beta; α-syn, α-synuclein; CI, confidence interval; GFAP, glial fibrillary acidic protein; IL-6, interleukin-6; NfL, neurofilament light; pTau181/217, phosphorylated tau at threonine 181/217; S100B, calcium-binding protein B; sTREM2, soluble triggering receptor expressed on myeloid cells 2; tTau, total tau; YKL 40, chitinase-3-like protein 1.

**Fig. 7:**
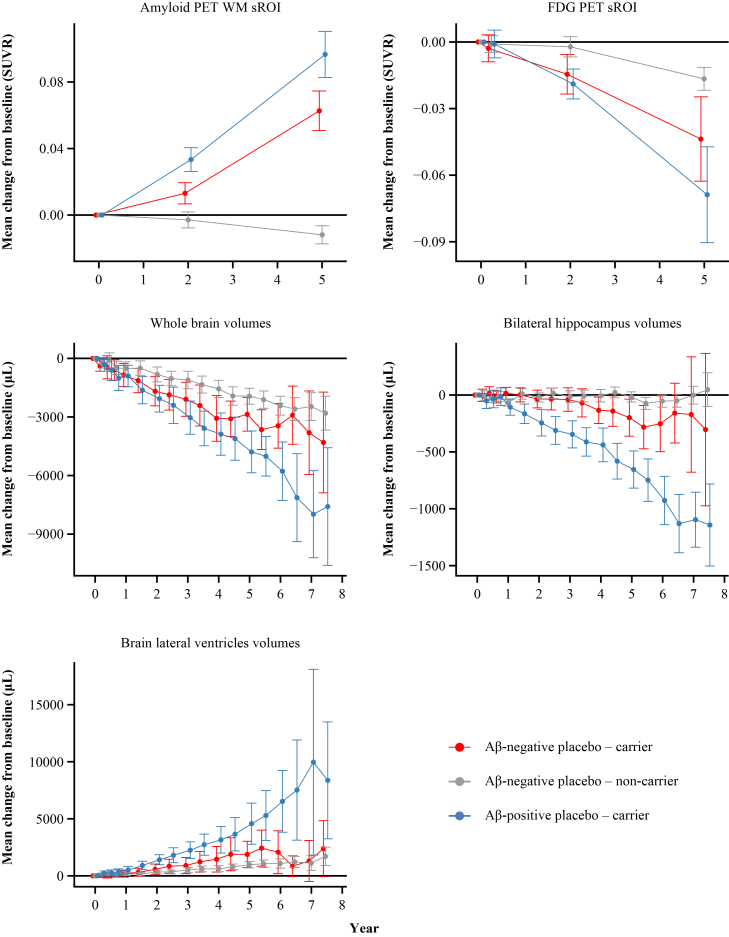
Longitudinal trajectories for imaging biomarker changes in placebo-treated mutation carriers with a positive or negative baseline amyloid PET scan and non-carriers, all of whom had a negative baseline amyloid PET scan. A positive amyloid PET scan is defined as ≥24.3 CL. Point estimates of unadjusted mean change from baseline with 95% CI are shown. sROI refers to SUVRs in an empirically pre-established statistical ROI that has been shown to preferentially decline in AD.^22^ Aβ, amyloid-beta; AD, Alzheimer’s disease; CI, confidence interval; FDG, fluorodeoxyglucose; PET, positron emission tomography; sROI, statistical region of interest; SUVR, standardised uptake value ratio; WM, white matter.

**Fig. 8:**
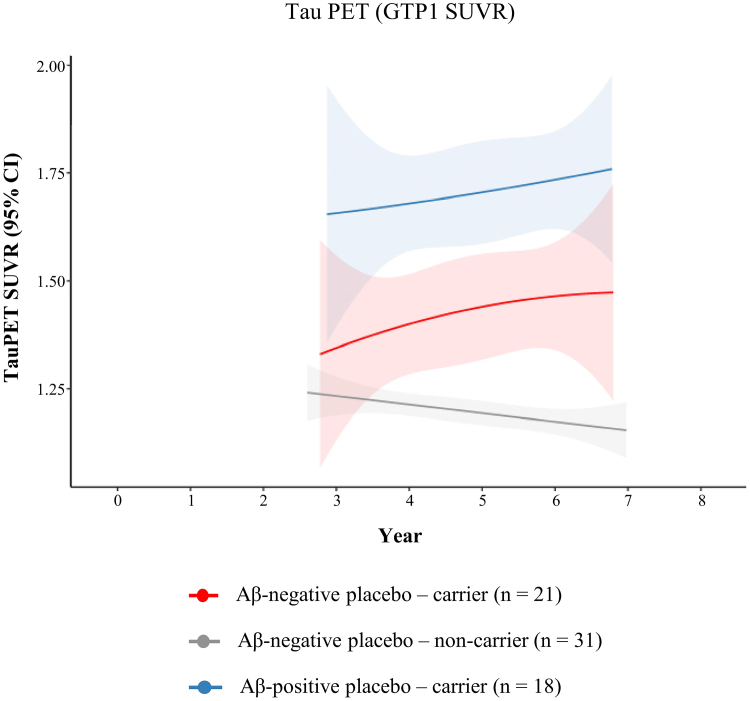
Longitudinal tau PET biomarker changes in placebo-treated mutation carriers with a positive or negative baseline amyloid PET scan and non-carriers, all of whom had a negative baseline amyloid PET scan. A positive amyloid PET scan is defined as ≥24.3 CL. Trend lines and 95% CIs were generated via LOESS smoothing to represent the average longitudinal trajectory within each cohort despite inconsistent measurement timing. Aβ, amyloid-beta; CI, confidence interval; GTP1, Genentech Tau Probe 1; PET, positron emission tomography; SUVR, standardised uptake value ratio.

**Fig. 9:**
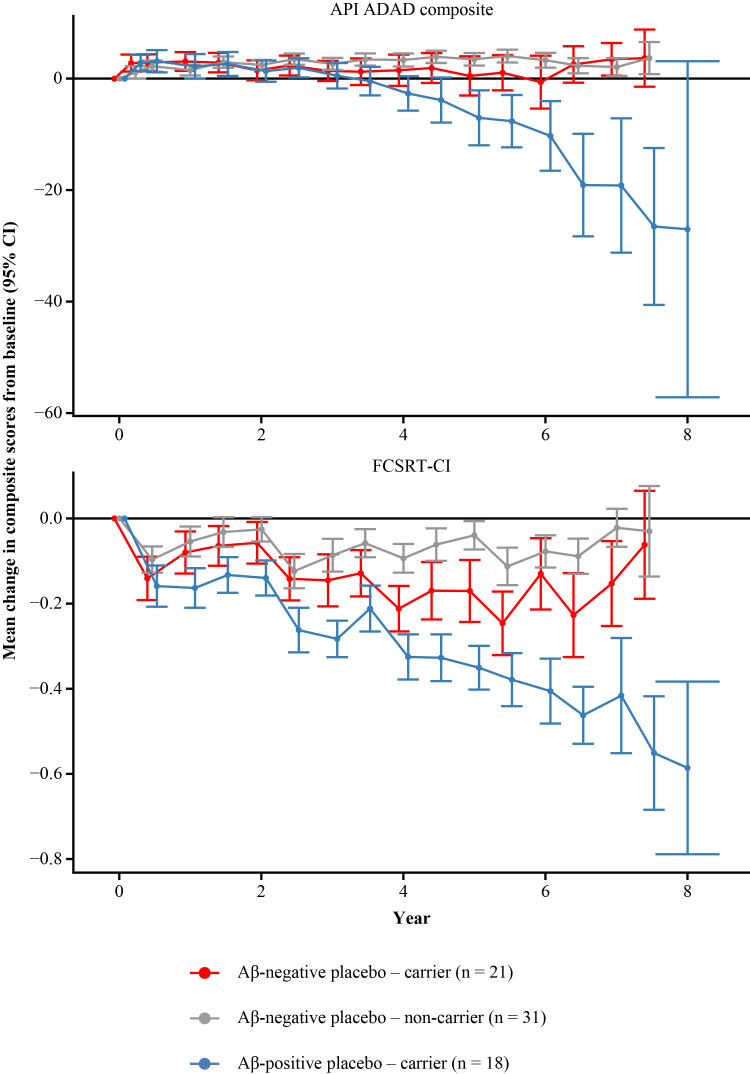
Trajectory of cognitive changes in placebo-treated mutation carriers with a positive or negative baseline amyloid PET scan and non-carriers, all of whom had a negative baseline amyloid PET scan. A positive amyloid PET scan is defined as ≥24.3 CL. Aβ, amyloid-beta; API ADAD, Alzheimer’s Prevention Initiative Autosomal Dominant Alzheimer’s Disease; CI, confidence interval; FCSRT, Free and Cued Selective Reminding Test.

Fig. 5, Fig. 6, Fig. 7, Fig. 8 present longitudinal trajectories for plasma biomarkers (Fig. 5), CSF biomarkers (Fig. 6), amyloid, tau, FDG PET, and volumetric MRI measurements (Fig. 7), tau PET measurements (Fig. 8), and the API ADAD composite and FCSRT-CI scores (Fig. 9) in placebo-treated amyloid PET-positive mutation carriers (i.e., those with baseline amyloid PET scans ≥ 24.3 CL), amyloid PET-negative carriers, and non-carriers (all of whom were amyloid PET-negative). Based on visual assessment of non-overlapping 95% CIs, placebo-treated carriers, compared with non-carriers, showed descriptively greater increases in mean cortical amyloid PET, entorhinal cortex temporal tau PET, MRI brain shrinkage, and biomarker measurements including CSF pTau181, tTau, NfL, YKL 40, neurogranin, and S100B, and plasma pTau217, pTau181, NfL, GFAP, and sTREM2. They also exhibited descriptively greater declines in CSF total Aβ42, with no visually apparent longitudinal differences observed for other biomarker measurements. Based on the plotted longitudinal trajectories, amyloid PET-positive mutation carriers demonstrated the steepest mean increases in amyloid and tau PET measurements and the steepest mean declines in FDG PET and MRI brain volume measurements relative to the other groups.

Based on the rates of MCI or dementia progression (Supplementary Table S1), we estimate that it would take 90 30–60-year-old amyloid PET-positive mutation carriers without cognitive impairment per arm (95% CI 57–194) to detect a 50% reduction in progression to an adjudicated diagnosis of MCI or dementia with 80% power in a 60-month placebo-controlled secondary prevention trial. We estimate that it would take 361 30–60-year-old amyloid PET-negative mutation carriers without cognitive impairment per arm (95% CI 187–3030) to detect a 50% reduction in progression to MCI or dementia, which is not feasible; and approximately 33 amyloid PET-negative mutation carriers per arm (95% CI 20–58) to detect a 75% reduction in progression to a positive amyloid PET scan in a 60-month placebo-controlled primary prevention trial. Required sample size estimates based on each of the dual primary endpoints for this study are provided in Supplementary Table S2.

### *Post hoc* CSF oligomeric findings

As previously noted, crenezumab treatment was distinguished from placebo by significantly greater CSF oAβ concentrations in mutation carriers in a *post hoc* analysis using a recently developed assay of relatively small oligomeric species. However, the mutation carrier and non-carrier groups did not differ significantly in their baseline CSF measurements (Table 2) or placebo-related longitudinal changes (Fig. 6).

## Discussion

This report describes baseline and placebo-related longitudinal biomarker, cognitive, and clinical findings in *PSEN1 E280A* mutation carriers and non-carriers without cognitive impairment from the API ADAD Colombia Trial. It provides new information about the biomarker changes associated with the detection and tracking of ADAD, the subsequent risk of cognitive impairment in carriers with and without biomarker evidence of Aβ plaques, and the number of Aβ-positive and negative carriers needed to evaluate primary and secondary ADAD prevention therapies using clinical or biomarker endpoints.

At baseline, mutation carriers without cognitive impairment had an average of 29.3 CL in their amyloid PET scans, slightly greater than the 24.3 CL threshold used to define the presence of moderately frequent neuritic Aβ plaques. Despite their relatively early preclinical ADAD stage, carriers were distinguished from non-carriers at baseline by PET, CSF, and/or plasma biomarker evidence of Aβ plaque burden, Aβ-mediated tau pathophysiology, neurodegeneration, and neuroinflammation. In addition to expected differences in baseline amyloid PET, FDG PET, and CSF total Aβ42, pTau181, total tau, and NfL measurements, the carriers had elevated CSF levels of neurogranin (a measure of post-synaptic neurodegeneration), GFAP, and α-syn concentrations (a possible indicator of Lewy bodies/neurites).

55% of the carriers had a positive baseline amyloid PET scan and 45% did not. The inclusion of those with and without a positive amyloid PET scan enabled exploration of the differential impact of the investigational drug therapy in people with no cognitive impairment with or without biomarker evidence of moderately frequent neuritic Aβ plaques (i.e., in the secondary and primary prevention of ADAD^10^). At the same time, the inclusion of a large number of amyloid PET-negative mutation carriers may have reduced statistical power to detect significant treatment effects.

The accuracy of a new fully automated pTau217 assay was also investigated, to distinguish between ADAD mutation carriers without cognitive impairment, with and without moderately frequent neuritic Aβ plaques using an amyloid PET ‘standard of truth’ and the value of using amyloid PET and plasma pTau217 measurements to inform the mutation carriers’ subsequent rates of progression to MCI or dementia.

While Roche’s second-generation plasma pTau217 assay had a moderately high accuracy in discriminating between amyloid PET-positive and PET-negative ADAD mutation carriers without cognitive impairment (AUC 0.78 [95% CI: 0.70, 0.85]; Supplementary Fig. S1), it was notably less accurate than in older adults without cognitive impairment and a known mutation with and without PET evidence of amyloid plaques (AUC 0.94 [95% CI: 0.92, 0.96]).^16^ This possible difference in the performance of plasma pTau217 relative to florbetapir PET could reflect limitations in the use of amyloid PET as a ‘standard of truth’ for the assessment of neuritic Aβ plaque deposition in ADAD. These limitations include the possibility of greater accumulation in the cerebellar reference region used to compute baseline cerebral-to-reference region SUVRs and related CL values, a greater proportion of diffuse to neuritic Aβ plaques, the possibility of greater regional and individual variability in this proportion, and a smaller number of ADAD cases and age-matched controls to assess the performance of amyloid PET compared with the density of neuritic Aβ plaques in antemortem–postmortem comparison studies.^28^^,^^29^

In addition to cerebellar Aβ plaque pathology, some brain donors with the PSEN1 E280A mutation have also reported cerebellar pTau pathology in the clinical stages of ADAD.^30^ For that reason, we cannot exclude the possibility that our tau PET measurements might slightly underestimate tau tangle pathology in the clinical and even preclinical stages of ADAD. We continue to investigate the impact of different (e.g., cerebellar, pontine, and white matter) reference regions on the detection and tracking of preclinical ADAD.

Categorising mutation carriers without impairment based on their amyloid PET CL or plasma pTau217 tertiles, positive or negative amyloid PET scans, or high, intermediate, or low plasma pTau217 concentrations, baseline amyloid PET and plasma pTau217 measurements were highly informative in predicting subsequent rates of clinical progression. 32.9% and 6.8% of amyloid PET-positive and -negative carriers, respectively, 36.5%, 13.2%, and 0.0% of pTau217-positive, -intermediate, and -negative carriers, respectively, and no non-carriers developed MCI or dementia over the next 5 years. The percentages of younger adult ADAD mutation carriers in the highest amyloid PET and plasma pTau tertiles who progressed to MCI or dementia within 5 years were comparable to those reported in older adults without cognitive impairment at differential risk for the clinical onset of late-onset AD.^31^ Tertile-based groupings are highly dependent on sample distribution. The tertiles for amyloid PET CL in the A4 study were <46.1, 46.1–77.2, and >77.2 CL,^31^ compared with <16.5, 16.5–39.0, and >39.0 CL in mutation carriers in API. The lower tertile values in the API ADAD study may reflect the younger average age of our study population and that the population did not show cognitive impairment at baseline.

*Post hoc* analyses utilising a new CSF oAβ assay demonstrated higher CSF oAβ concentrations in the crenezumab than placebo carrier group, possibly due to reduced clearance of the antibody-bound oAβ species.^10^ Since the carriers did not differ from non-carriers in their baseline measurements or longitudinal changes, the role of these oligomers in the development, treatment, and prevention of AD remains to be clarified.

To illustrate how the trial data can inform the design and sample size of future prevention trials, we estimate that 90 30–60-year-old amyloid PET-positive mutation carriers per arm would be required to detect a potential ADAD prevention therapy’s ability to reduce the onset of cognitive impairment by 50% in a 60-month secondary prevention trial with adequate statistical power. While 361 amyloid-PET negative mutation carriers per arm would be required to evaluate its ability to reduce the onset of cognitive impairment by 50% in a 5-year primary prevention trial, approximately 33 amyloid PET-negative mutation carriers per arm (95% CI 20–58) would be needed to detect a 75% reduction in progression to a positive amyloid PET scan. Since the 5-year attrition rate in this study was remarkably low and since the reason for attrition was accounted for in every participant, these estimates were not confounded by differential survivor bias, another study strength. If ongoing secondary prevention trials demonstrate a clinical benefit, it may be possible to accelerate the evaluation and potential approval of a primary prevention therapy using a treatment’s impact on progression to biomarker evidence of Aβ positivity as a surrogate endpoint.

While the investigational drug in the API ADAD Colombia Trial failed to demonstrate a clinical benefit, the trial introduced new paradigms to accelerate the evaluation and potential approval of prevention therapies and has given the field a realistic chance to find the first effective AD prevention therapies within the next few years.^32^ Soon after the API ADAD Colombia Trial was announced, API, the Dominantly Inherited Alzheimer’s Network (DIAN), the Alzheimer’s Clinical Trial Consortium, and pharmaceutical company leaders began to develop other prevention trials, introduce new participant engagement, prevention research and resource-sharing programmes, and make critical contributions to the development of effective AD prevention therapies. The Collaboration for Alzheimer’s Prevention, which included representatives from the FDA, NIH, advocacy and philanthropic organisations, and the academic prevention programmes began to exchange research and resource-sharing ideas and consider best practices, lessons learnt, and evidence that might inform the approval of prevention therapies.^6^^,^^7^

Researchers recently used data from the DIAN to estimate the number of ADAD mutation carriers within 15 years of the estimated age of cognitive impairment needed to detect treatment effects using a wider range of PET, MRI, CSF, cognitive, and clinical endpoints, but without stratification for those who were amyloid plaque positive or negative at baseline.^33^ Here, we estimated the number of amyloid positive or negative PSEN1 E280A mutation carriers needed to detect treatment effects on several cognitive and clinical endpoints and on progression to amyloid PET positivity in a primary prevention trial. Data and samples from this study will provide a chance for researchers to estimate sample sizes needed for ADAD prevention trials in amyloid positive and negative mutation carriers using different biomarkers and clinical endpoints, and treatment durations.

The API ADAD Colombia Trial’s baseline and placebo-related longitudinal findings continue to advance the detection, tracking, diagnosis, study, treatment, and prevention of AD. For example, the trial data and samples may be used to consider the differential effects of crenezumab in mutation carriers without cognitive impairment with and without biomarker evidence of AD, assess the risk of subsequent cognitive decline and clinical progression in these ADAD sub-groups, and estimate the number of carriers needed to evaluate potential secondary and primary ADAD prevention therapies with adequate statistical power using different clinical, cognitive, and biomarker endpoints. A shared resource of trial data and biological samples is now available by request to support this endeavour.

## Contributors

All authors provided substantial contributions to the conception, design, analysis, and interpretation of the study. The first draft of the manuscript was written by TB and EMR. PNT, KMS, NH, JBL, and EMR directly accessed and verified the underlying data reported in the manuscript. Statistical analyses were performed by DH, VG, and YS. All authors provided their input to critically revise the first and second drafts of the manuscript for important intellectual content, approved the final version of the manuscript to be published, and had full access to all data in the study. Medical writing support for the development of the penultimate and subsequent manuscript draft was provided by Rachel Johnson, PhD, of Nucleus Global, funded by F. Hoffmann-La Roche Ltd.

## Data sharing statement

The study protocol and statistical analysis plan are available in the supplement of a separate report^10^ or https://clinicaltrials.gov/study/NCT01998841. Banner and Roche will make the trial data and biological samples available 18 months after the end of the study. Clinical data and trial imaging will be available through the University of Southern California Laboratory of Neuroimaging (USC LONI) at: https://loni.usc.edu/research/projects. Residual study biomaterial will be accessible by request through the Indiana University National Cellular Repository for Alzheimer’s Disease (NCRAD) at: https://www.ncrad.org/accessing_data.html. Data and samples housed at LONI and NCRAD are harmonised to enable analyses leveraging multiple study resources. Inquiries regarding study data and biomaterial, including access requests, may also be made directly through: APIData@bannerhealth.com.

## Declaration of interests

**TB** is a full-time employee of F. Hoffmann-La Roche Ltd and Genentech, Inc., a member of the Roche Group, and owns stock in F. Hoffmann-La Roche Ltd. **FL** received grants from the NIH, NIA, Roche, MSD, Biogen, and Tau Consortium. **SRR** was a full-time employee of Neuroscience Group of Antioquia, University of Antioquia, at the time of this work; she is currently employed at IQVIA; IQVIA had no involvement in this study. **CS**, **DC**, **DH**, **MD**, and **VP** are full-time employees of Genentech, Inc., a member of the Roche Group, and own stock in F. Hoffmann-La Roche Ltd. JN was a full-time employee of Genentech Inc. at the time of this study. **HP** has received relevant grants from the National Institute of Health (NIH; R01AG059390, RF1AG054617), and the National Institute on Aging (NIA; R01 AG054671). **KMS** was a full-time employee of Genentech, Inc., a member of the Roche Group, at the time of the study and owns stock in F. Hoffmann-La Roche Ltd. **GK** is a full-time employee of Roche Diagnostics GmbH, Germany. **NJA** has received consulting fees from AbbVie, Athria, ImaginationL and LLC, MapLight Therapeutics, SpearBio, Neurogen Biomarking, Quanterix, TauRx, Eli-Lilly, Roche Dx, Beckman Coulter, Janssen, Bristol Myers Squibb; honoraria for lectures, presentations, speakers bureaus, manuscript writing, or educational events from Alamar Biosciences, Biogen, Eli-Lilly, Quanterix, VJDementia, Beckman Coulter; has participated in advisory boards for Biogen, Bristol Myers Squibb New Amsterdam, Janssen, Roche, and TauRx; and discloses a patent application related to methods for remote blood collection and extraction and analysis of neuro biomarkers. **DJS** is supported by relevant grants from the NIA and National Institute of Neurological Diseases and Stroke. He has received consulting fees and support for attending meetings (with associated travel support), and stock options (not exercised) from Prothena Biosciences, and honoraria from the Duke University Medical School Department of Neurology (with travel support), University of Denver, and the State University of New York at Stoneybrook. He has also participated on a data safety monitoring board for Roche and a safety advisory board for Eisai. **RCA** is supported by relevant grants R01 AG058468, R01 AG055444, and R01 AG074983 from the NIA. He is one of the leaders of the Alzheimer’s Prevention Initiative (API), which is collaborating with Eli Lilly and Roche in its ongoing and planned AD prevention trials. He is also a compensated scientific advisor to Cenna, Lundbeck, Novartis, PRInnovations, Vigil Neuro and DSMB member for ImmunoBrain. **YTQ** serves as consultant for Biogen and reports receiving grants from the NIA, NIH, and NINDS, the Alzheimer’s Association, and Massachusetts General Hospital Executive Committee on Research, unrelated to this study. **YS** discloses grant support from the NIH and State of Arizona. **RSD** was an employee of F. Hoffmann-La Roche Ltd and Genentech Inc., part of F. Hoffmann-La Roche Ltd, at the time of this work and may own stock/stock options in F. Hoffmann-La Roche Ltd; she has no additional potential conflicts of interest to disclose. **JBL** served as a consultant to Biogen and Denovo Biopharma. She reports receiving grants from the NIA (P30AG072980). **PNT** reports receiving grants from the NIA (UF1AG046150, RF1 AG041705-01A1, R01 AG055444, and 1R01AG058468), the Banner Alzheimer’s Foundation, and the NOMIS Foundation, as well as receiving consulting fees from AbbVie, AC Immune, Acadia, Athira, Axsome, Biogen, BioXcel, Bristol Myers Squibb, Cognition Therapeutics, Corium, Cognito, Cortexyme, CuraSen, Eisai, Genentech, ImmunoBrain, Janssen, Lundbeck, MapLight, Merck and Co., Novartis, Novo Nordisk, ONO Pharmaceuticals, Otsuka & Astex, Roche, Syneos, and T3D Therapeutics. **EMR** is a principal investigator of several NIH, foundation, and Arizona grants; inventor of a 2005 patent to accelerate the evaluation of Alzheimer’s disease prevention therapies using biomarker endpoints in people at genetic or biomarker risk; a compensated scientific advisor to Alzheon, Aural Analytics, Beren Therapeutics, Cognition Therapeutics, Denali Therapeutics, Enigma Diagnostics, New Amsterdam Therapeutics, Retromer Therapeutics, and Vaxxinity; and a co-founder and advisor to ALZPath. The remaining authors have no conflicts of interest to disclose.
