# Supplementary material for: Baseline and placebo-related imaging, cerebrospinal fluid, plasma biomarker, and cognitive findings in unimpaired PSEN1 E280A mutation carriers and non-carriers in the Alzheimer’s Prevention Initiative Autosomal Dominant Alzheimer’s Disease Colombia Trial
Source: eBioMedicine. 2026 Jul 17;130:106390. doi: 10.1016/j.ebiom.2026.106390 (PMC13400256; doi:10.1016/j.ebiom.2026.106390)
Supplement: Supplementary Appendix [file mmc1.docx]

**SUPPLEMENTARY APPENDIX**

**Supplementary materials for: Baseline and placebo-related imaging, cerebrospinal fluid, plasma biomarker, and cognitive findings in unimpaired *PSEN1* *E280A* mutation carriers and non-carriers in the Alzheimer’s Prevention Initiative Autosomal Dominant Alzheimer’s Disease Colombia Trial**

**Contents**

[API ADAD Colombia trial investigators 2](#_Toc231827997)

[Supplementary methods 3](#_Toc231827998)

[Study design and participants 3](#_Toc231827999)

[Study drug administration and formulation 3](#_Toc231828000)

[Cerebrospinal fluid biomarker collection and analysis 3](#_Toc231828001)

[Blood biomarker collection and analysis 3](#_Toc231828002)

[Oligomeric amyloid-beta collection and analysis 3](#_Toc231828003)

[Methodology for two-threshold pTau217 strategy 4](#_Toc231828004)

[Sample size estimates for clinical and amyloid PET progression outcomes 4](#_Toc231828005)

[Supplementary tables 5](#_Toc231828006)

[Supplementary table 1 Cumulative clinical progression to MCI or dementia and corresponding sample size estimates for hypothetical placebo-controlled prevention trials, stratified by baseline amyloid PET status. 5](#_Toc231828007)

[Supplementary table 2 Sample size estimates to detect a 50% slowing of cognitive decline on the API ADAD composite and FCSRT-CI over 260 weeks in a placebo-controlled prevention trial. 6](#_Toc231828008)

[Supplementary figures 7](#_Toc231828009)

[Supplementary fig. 1 Graphical representation of the ROC 7](#_Toc231828010)

[References 8](#_Toc231828011)

[Equitable Partnership Declaration 9](#_Toc231828012)

API ADAD Colombia trial investigators

Francisco Lopera *(Neuroscience Group of Antioquia, University of Antioquia, Medellín, Colombia)*, Silvia Ríos-Romenets *(Neuroscience Group of Antioquia, University of Antioquia, Medellín, Colombia)*, Margarita Giraldo-Chica *(Neuroscience Group of Antioquia, University of Antioquia, Medellín, Colombia)*, Natalia Acosta-Baena *(Neuroscience Group of Antioquia, University of Antioquia, Medellín, Colombia)*, Claudia Ramos *(Neuroscience Group of Antioquia, University of Antioquia, Medellín, Colombia)*, Claudia Aponte *(Neuroscience Group of Antioquia, University of Antioquia, Medellín, Colombia)*, Laureano Mesta *(Neuroscience Group of Antioquia, University of Antioquia, Medellín, Colombia)*, Ramiro Martinez *(Neuroscience Group of Antioquia, University of Antioquia, Medellín, Colombia),* Alejandro Espinosa *(Neuroscience Group of Antioquia, University of Antioquia, Medellín, Colombia and Hospital San Juan de Dios de Yarumal, Antioquia, Colombia),* Carlos Tobón *(Universidad de Antioquia, School of Medicine, Medellín, Colombia)*, Gregorio Sánchez *(Cardiomet Cequin, Universidad del Quindío, and San Juan de Dios Hospital, Armenia, Colombia)* Mario Muñoz Collazos *(Marly’s Clinic, Bogotá, Colombia)*

# Supplementary methods

## Study design and participants

Trial eligibility criteria included *PSEN1* *E280A* kindred membership, age 30–60 years, being without cognitive impairment and medically stable, having a study partner, no history of treatment for Alzheimer’s disease, and a body weight of 45–120 kg. Participants’ sex was obtained from their identification document (Cédula de Ciudadanía) in the Registry and confirmed with self-report (there were no discrepancies). Participants were included irrespective of whether they had baseline florbetapir positron emission tomography (PET) evidence of amyloid-beta (Aβ) plaques, defined by a mean cortical-to-cerebellar standardised uptake value ratio (SUVR) of 1·10 (24·3 centiloids).^1^ Enrolled participants were followed until the last randomised participant received 260 weeks of treatment, whereby the first and last participants enrolled concluded the trial at the same time; treatment periods ranged from 5 to nearly 8 years. Assessments were performed as indicated in a protocol summary published previously.^2^

## Study drug administration and formulation

Crenezumab was initially administered at 300 mg subcutaneously (SC) every 2 weeks; the dose was increased to 720 mg SC every 2 weeks; an optional dose increase to 60 mg/kg intravenously (IV) every 4 weeks was offered. Once participants received IV dosing, they were not allowed to revert to SC dosing to maximise drug exposure in the most consistent way possible. Crenezumab was formulated as 180 mg/mL crenezumab in 200 mM arginine succinate, 0·05% (w/v) polysorbate 20, pH 5·5, and supplied in 6 mL glass vials as a 4 mL liquid. The placebo was formulated and supplied using the same method but without the active drug. All participants were allowed standard‑of-care medications (e.g., memantine and acetylcholinesterase inhibitors) if they developed symptomatic Alzheimer’s disease.

## Cerebrospinal fluid biomarker collection and analysis

At each time point, approximately 22 mL of cerebrospinal fluid (CSF) was collected into 10 mL syringes (standard hospital supply) using a 24G Sprotte needle (order number 121151-30A, Pajunk GmbH, Germany) by lumbar puncture at L4/L5 (the first 1–2 mL of CSF was discarded if the CSF was pink). 0·5 mL of the CSF from the first syringe was distributed into 1·5 mL LoBind^®^ PP tubes (order number 022431081, Eppendorf, Germany) using a 3·5 mL transfer pipette (order number 86.1171.002, Sarstedt AG, Nümbrecht, Germany) and immediately frozen and shipped on dry ice the day of sample collection. CSF was stored at the central lab at −70°C and thawed at room temperature for 1 hour before measurement. Baseline and follow-up samples of the same participant were measured in one batch using single reagent lots at the end of the study. Effects of differences of sample time in the freezer were not evaluated.

## Blood biomarker collection and analysis

Approximately 6 mL whole blood was collected into a 6 mL K2 EDTA primary collection tube (order number 367863, BD, Allschwil, Switzerland). The primary collection tube was inverted gently 8–10 times by hand to ensure the anticoagulant was mixed well with the blood. Within 60 minutes of blood collection, the blood was centrifuged at 1500 g for 15 minutes at room temperature, and 0·5 mL of supernatant was dispensed into 4 mL PP cryovials (order number 430490, Corning, Root, Switzerland) and immediately frozen and stored at –20°C for screening and baseline and at −70°C for all subsequent samples. Samples were shipped on dry ice to the central lab for testing and long-term storage was kept at −70°C. All baseline and follow-up samples of the same participant were analysed in one batch using a single reagent lot at the end of the study.

## Oligomeric amyloid-beta collection and analysis

Biotinylated capture monoclonal antibody 71A1 was conjugated to streptavidin magnetic particles (MPs) (Dynabeads^TM^ MyOne^TM^, Thermo Fisher Scientific) at a ratio of 12·5 μg biotinylated 71A1 antibody per mg of MPs (EMD Millipore). MPs with bound capture antibody were diluted to 50 μg/mL in Aβ-Oligomer Assay Buffer (Tris buffer; 50 mM Tris, 150 mM NaCl, pH 7·6 with 1% Triton X-100, 0·0005% [w/v] d-des-thio-biotin and 0·1% bovine serum albumin). A volume of 50 μL of this suspension was added to 150 μL of sample, standard, or blank, and incubated at 600 rpm on a shaking incubator at 25°C for 2 h. MPs were isolated using a magnet, and unbound material was removed by washing with 1x SMC^TM^ wash buffer (EMD Millipore) using a BioTek plate washer. Fluorescently labelled (Alexa-647 dye) anti-Aβ detection antibody 3D6 (20 μL, 200 ng/mL) was added to each well. MPs bearing the antibody–oligomer Aβ sandwich were then incubated with agitation using a Jitterbug shaker (Boekel, Feasterville, PA, USA) for 1 hour at 25°C. Unbound detection reagent was removed by washing (four times) with SMC^TM^ wash buffer. Wash buffer was removed by aspiration, and fluorescently labelled 3D6 detection antibody was released from the beads by shaking in Elution Buffer B (EMD Millipore, Cat # 02-0297-00, 11·5 μL/well) for 10 minutes at 25°C. A volume of 11 μL of each eluate were then transferred to the wells of a clean 96‑well plate containing Neutralisation Buffer D (EMD Millipore, Cat # 02-0368-00, 11 μL/well). The neutralised sample (20 μL/well) was then transferred to a black 386-well plate (Aurora) and read by the SMCxPRO instrument with 642 nm laser. The lower limit of reliable quantification was defined as the lowest back interpolated standard that provides a signal two-fold the background with a percentage of recovery calculated between 80% and 100% and coefficient of variance ≤ 20%.

## Methodology for two-threshold pTau217 strategy

Plasma pTau217 thresholds were determined using data from 616 participants (mean age [SD] 68 ± 8 years; 543 without cognitive impairment, 73 with cognitive impairment [MCI/dementia]) enrolled in the Wisconsin Registry for Alzheimer's Prevention (WRAP).^3^ Plasma p-tau217, measured on the Roche cobas e801 platform using a Research Use Only (RUO) assay, was evaluated for its ability to predict amyloid PET positivity, defined as > 24·1 Centiloids. Multiple pTau217 cutoff values were assessed, and the threshold achieving sensitivity and specificity closest to approximately 95% each was selected. This cutoff was subsequently applied to stratify individuals into low, intermediate, and high pTau217 groups, yielding a positive predictive value of 85% and a negative predictive value of 98% in the WRAP cohort. Overall discriminative accuracy for amyloid positivity in this cohort was 95%.

## Sample size estimates for clinical and amyloid PET progression outcomes

Cumulative progression probabilities to mild cognitive impairment (MCI) or dementia were obtained from Kaplan–Meier analyses at yearly time points within baseline amyloid-positive and amyloid-negative subgroups. Because clinical progression risk was summarised at fixed follow-up time points, a binomial proportion framework was used for sample size estimation. Hypothetical treatment effects were modelled as relative reductions in progression probability of 50% at each time point. For cumulative progression estimates, 95% confidence intervals were derived from the Kaplan–Meier survival function and transformed to the cumulative incidence scale; these bounds were propagated through the same sample size calculation framework to obtain corresponding ranges for the estimated sample size per treatment arm. Required sample sizes per treatment arm were calculated using a two-sided test for equality of proportions with a significance level of 0·05, 80% statistical power, and equal allocation between treatment groups.

The sample size estimates for both the clinical progression and amyloid PET progression were calculated with R version 4.4.3 using the power.prop.test for two-group comparisons of proportions with a two-sided α = 0.05 and 80% power.

For exploratory estimation of sample size requirements for prevention of amyloid PET progression, longitudinal participant-level amyloid PET measurements in baseline amyloid-negative mutation carriers were analysed using linear mixed-effects models to estimate individual biomarker trajectories and predicted time to crossing of the predefined amyloid positivity threshold (24·3 CL) over a 60-month period. The predicted cumulative probability of amyloid PET conversion was then used within the same binomial sample size framework to estimate the number of participants required per treatment arm under a hypothetical 75% relative reduction in amyloid progression risk. Uncertainty in the predicted conversion probability was quantified using exact binomial confidence intervals and propagated through the corresponding sample size calculations.

# Supplementary tables

## Supplementary table 1 Cumulative clinical progression to MCI or dementia and corresponding sample size estimates for hypothetical placebo-controlled prevention trials, stratified by baseline amyloid PET status.

| **Amyloid status at baseline** | **Year** | **Cumulative progression in placebo arm**  **(95% CI)^a^** | **Estimated n per arm to detect 50% reduction in progression**  **(95% CI)^b^** |
| --- | --- | --- | --- |
| Amyloid-PET positive | 1 | 0·024  (0·000, 0·070) | 1895  (632, 632) |
|  | 2 | 0·073  (0·000, 0·150) | 608  (279, 279) |
|  | 3 | 0·098  (0·002, 0·185) | 443  (218, 23650) |
|  | 4 | 0·249  (0·102, 0·371) | 153  (91, 425) |
|  | **5** | **0**·**374**  **(0·205, 0·507)** | **90**  **(57, 194)** |
| Amyloid-PET negative | 1 | 0·023  (0·000, 0·067) | 1989  (664, 664) |
|  | 2 | 0·023  (0·000, 0·067) | 1989  (664, 664) |
|  | 3 | 0·023  (0·000, 0·067) | 1989  (664, 664) |
|  | 4 | 0·071  (0·000, 0·145) | 628  (289, 289) |
|  | **5** | **0**·**119**  **(0·015, 0·211)** | **361**  **(187, 3030)** |

^a^95% confidence intervals were derived from the Kaplan–Meier survival function and transformed to the cumulative incidence scale; these bounds were propagated through the same sample size calculation framework to obtain corresponding ranges for the estimated sample size per treatment arm.

^b^Sample size estimates refer to the number of participants who complete the trial, assume a two-sided α of 0·05, 80% power, equal allocation between treatment groups, and hypothetical relative reductions in clinical progression probability of 50% at the specified follow-up time point. As sample size depends non-linearly on the assumed progression probability, confidence intervals for sample size estimates may be asymmetric and may not include the point estimate when progression risks are very low.

CI, confidence interval; MCI, mild cognitive impairment; PET, positron emission tomography.

## Supplementary table 2 Sample size estimates to detect a 50% slowing of cognitive decline on the API ADAD composite and FCSRT-CI over 260 weeks in a placebo-controlled prevention trial.

| **Endpoint** | **Amyloid status at baseline** | **Week** | **n (placebo arm)** | **Mean change from baseline (95% CI)^a^** | **Estimated n per arm**  **(95% CI)^b^** |
| --- | --- | --- | --- | --- | --- |
| **API ADAD composite** | Positive | 260 | 39 | -7·064  (-12·210, -1·917) | 318  (107–4305) |
|  | Negative | 260 | 40 | 0·515  (-3·097, 4·128) | 30189  (471–Inf) |
| **FCSRT-CI** | Positive | 260 | 39 | -0·351  (-0·403, -0·298) | 14  (11–19) |
|  | Negative | 260 | 39 | -0·171  (-0·247, -0·096) | 117  (57–375) |

^a^Mean change 95% CIs computed as t-intervals; the resulting CI bounds were propagated through the two-sample continuous-outcome power calculation to provide an n/arm range.

^b^Sample size estimates refer to the number of participants who complete the trial (range based on 95% CI of mean change).

API ADAD composite, Alzheimer’s Prevention Initiative preclinical autosomal-dominant Alzheimer’s disease composite test score; CI, confidence interval; FCSRT-CI, Free and Cued Selective Reminding Test-Cueing Index; inf, infinity.

# Supplementary figures

## Supplementary fig. 1 Graphical representation of the ROC

**
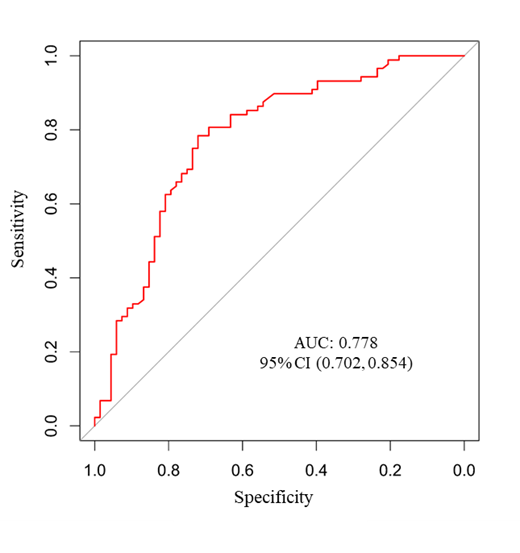
**

An AUC 0f 0.778 indicates the accuracy of plasma p-tau217 for discriminating between PSEN1 E280A mutation carriers without cognitive impairment with a positive vs negative amyloid PET scan, with amyloid positivity defined as > 24.1 Centiloids.

AUC, area under the curve; CI, confidence interval; ROC, receiver operating characteristic curve.

# References

1. Navitsky M, Joshi AD, Kennedy I, et al. Standardization of amyloid quantitation with florbetapir standardized uptake value ratios to the centiloid scale. *Alzheimers Dement*. 2018;14(12):1565-1571. <https://doi.org/10.1016/j.jalz.2018.06.1353>

2. Tariot PN, Lopera F, Langbaum JB, et al. The Alzheimer's Prevention Initiative Autosomal-Dominant Alzheimer's Disease Trial: a study of crenezumab versus placebo in preclinical PSEN1 E280A mutation carriers to evaluate efficacy and safety in the treatment of autosomal-dominant Alzheimer's disease, including a placebo-treated noncarrier cohort. *Alzheimer's & dementia (New York, NY)*. 2018;4150-160. <https://doi.org/10.1016/j.trci.2018.02.002>

3. Johnson SC, Koscik RL, Jonaitis EM, et al. The Wisconsin Registry for Alzheimer's Prevention: a review of findings and current directions. *Alzheimer's & Dementia: Diagnosis, Assessment & Disease Monitoring*. 2018;10130-142.

# Equitable Partnership Declaration

If any questions do not apply to your study, please indicate “N/A” for “not applicable.
For more information on how to complete this form see the Information for Authors document.

Researcher considerations

1. Please detail the involvement that researchers who are based in the country or countries of study had during a) study design; b) clinical study processes, such as processing blood samples, prescribing medication, or patient recruitment; c) data interpretation; and d) manuscript preparation, commenting on all aspects. If they were not involved in any of these aspects, please explain why.

*This should include a thorough description of their leadership roles in the study. Are local researchers named in the author list or the acknowledgements, or are they not mentioned at all (and, if not, why)? Please also describe the involvement of early career researchers based in the location of the study. Some of this information might be repeated from the Contributors section in the manuscript. Note: we adhere to [ICMJE authorship criteria](https://www.icmje.org/recommendations/browse/roles-and-responsibilities/defining-the-role-of-authors-and-contributors.html) for naming authors on a paper.*

| **a) Study design:** Banner Alzheimer’s Institute, Genentech, Inc., F. Hoffmann-La Roche Ltd, and the Grupo de Neurociencias de Antioquia (GNA) at the University of Antioquia shared responsibility for study design; all listed authors (including investigators at the study site) provided substantial contributions to the study design; Colombian ADAD kindred members were also asked for their input during the selection of the investigational AD-modifying treatment (full details published previously: Tariot PN, et al. Alzheimers Dement 2018; 4: 150–60) |
| --- |
| **b) Clinical study processes:** All study investigators |
| **c) Data interpretation:** All study authors |
| **d) Manuscript preparation:** All study authors |

1. How was funding used to remunerate and enhance the skills of researchers in the countries of study? And how was funding used to improve research infrastructure at the study sites?

*Potentially effective investments into long-term skills and opportunities within local institutions could include training or mentorship in analytical techniques and manuscript writing, opportunities to lead all or specific aspects of the study, financial remuneration rather than requiring volunteers, and other professional development and educational opportunities.*

*Improvements to research infrastructure could include funding extended trial designs (eg, platform trials), establishment of long-term contracts for research staff, building research facilities, and setting up local control of funding allocation.*

| **Skills:** NIH, industry, and/or philanthropic funding was used to support, train and monitor the performance of all study investigators. |
| --- |
| **Research infrastructure:** NIH, industry, philanthropic, and/or local funding was used to develop the study, MRI, PET, and cyclotron resources needed to conduct this and related studies. |

1. How did you safeguard the researchers who implemented the study?

*Please describe how you guaranteed safe working conditions for study staff, including provision of appropriate personal protective equipment, protection from violence, and prevention of overworking.*

| Researchers implementing the API Colombia study were safeguarded through structured operational and institutional measures to ensure safe and sustainable working conditions. The study team included primary investigators and designated back-up personnel, allowing for appropriate workload distribution, continuity of study visits, and protection of scheduled vacation and rest periods. An established Occupational Health and Safety Program ensured compliance with local regulations, including the provision of appropriate personal protective equipment (PPE), staff training, and ongoing risk monitoring.  In addition, regular team coordination, clear operational procedures, and supervisory oversight were implemented to prevent excessive workload. Access to institutional well-being resources and psychosocial support was also available when needed, contributing to a safe, supportive, and resilient research environment. |
| --- |

*Benefits to the communities and regions of study*

1. How does the study address the research and policy priorities of its location?

*How were the local priorities determined and then used to inform the research question? Who decided which priorities to take forward? Which elements of the study address those priorities?*

| Banner Alzheimer’s Institute and GNA researchers worked closely with members or the Colombian kindred to understand their own research priorities, the kind of treatment they would value in terms of mechanism of action and safety data, and the importance of this work to members of their ADAD kindred and other families around the world. Philanthropic funds were also used to support a GNA-administered social plan that provided information, education, and support to members of the kindred. |
| --- |

1. How will research products be shared in the community of study?

*For instance, will you be providing written or oral layperson summaries for non-academic information sharing? Will study data be made available to institutions in the region(s) of study?* The Lancet Group *encourages authors to translate the summary (abstract) into relevant languages after paper editing; do you intend to translate your summary?*

| This was the world’s first prevention trial of an investigational disease-modifying treatment in cognitively unimpaired persons at high biological risk for cognitive impairment due to AD. The study generated international interest in the families and their role in the effort to find effective therapies for AD prevention. It was highlighted in the announcement of the National Plan to Address AD in 2012. The transparent working relationship between researchers and family members, one that embraced help and hope, led to a remarkable participation rate in the trial and continued interest in participating in future studies.  A $75M NIH-funded trial in this kindred is expected to begin within the next few months. In the trial, virtually all of the participants will receive initial treatment with an amyloid plaque-clearing antibody therapy that has been shown to benefit cognitively impaired persons with late-onset AD, and they will also receive investigational treatments that are intended to stave off further biomarker progression of the disease following amyloid clearance.  The families continue to benefit from our philanthropically supported GNA-administered social plan.  The initial study did not disclose a person’s genetic status, since there was no approved mechanism to do so in Colombia and the vast majority of ADAD kindred members preferred not to learn about their genetic study. During the study, we generated additional support to develop genetic counselling and risk disclosure procedures for family members who may be interested in learning their results in the future.  Throughout the study, annual meetings were held with participants of the clinical trial to share updates on the progress of the project. These meetings included information on results from other clinical trials involving the same investigational molecule worldwide, as well as findings from other Alzheimer’s disease trials. Participants were also informed about the final results of the study and the next steps for follow-up within the research group, including opportunities for participation in future prevention trials such as those described above. |
| --- |

1. How were individuals, communities, and environments protected from harm?
   1. *How did you ensure that sensitive patient data were handled safely and respectfully? Was there any potential for stigma or discrimination against participants arising from any of the procedures or outcomes of the study?*

| From the inception of the study, an Ethics and Cultural Sensitivities Committee, including researchers, ethicists, and members of the local community were involved in vetting all relevant issues related to the design and performance of the study, our genetic disclosure/non-disclosure strategy, and data and sample sharing policies. We routinely informed interested kindred members of our progress and solicited their input and advice whenever needed.  While this study introduced a precedent-setting commitment to share trial data and biological samples to have the greatest possible impact, policies and procedures were developed by API and other researchers in the Collaboration for Alzheimer’s Prevention (CAP) to minimize the risk that data, samples, figures and tables would inadvertently disclose a kindred member’s genetic risk. |
| --- |

- 1. *Might any of the tests be experienced as invasive or culturally insensitive?*

| Please see above. |
| --- |

- 1. *How did you determine that work was sensitive to traditions, restrictions, and considerations of all cultural and religious groups in the study population?*

| Please see above. |
| --- |

- 1. *Were biowaste and radioactive waste disposed of in accordance with local laws?*

| Yes: biowaste was disposed of safely and appropriately. We did not generate radioactive  waste. |
| --- |

- 1. *Were any structures built that would have impacted members of the community or the environment (such as handwashing facilities in a public space)? If so, how did you ensure that you had appropriate community buy-in?*

| Physical adaptations were implemented to ensure the proper conduct of the study and to support participant well-being. These included the provision and adjustment of patient bathrooms, handwashing stations at the entrance of the clinical area, lockers for personal belongings, and access to water dispensers for participants.  Hand hygiene campaigns were also conducted, and participants were routinely asked to wash their hands upon entering the clinical area. Visual aids illustrating the handwashing steps recommended by the WHO were displayed to reinforce these practices.  In addition, personal protective equipment (PPE) was provided when required, in accordance with local regulations and the specific procedures performed. |
| --- |

- 1. *How might the study have impacted existing health-care resources (such as staff workloads, use of equipment that is typically employed elsewhere, or reallocation of public funds)?*

| The study was designed and implemented in a way that minimized any negative impact on existing healthcare resources.  Regarding staff workload, a dedicated research team was assigned to the study, including primary and back-up personnel, which ensured that study-related activities did not interfere with routine clinical care or overburden existing healthcare staff. In terms of infrastructure and equipment, the study was conducted within research-designated facilities, and any required equipment was either specifically allocated to the study or managed to avoid disruption of standard clinical services. No essential clinical resources were diverted from patient care. From a financial perspective, study activities were supported by external research funding, and no public health funds were reallocated. On the contrary, the study contributed to strengthening local research capacity, including staff training, infrastructure improvements, and long-term institutional development.  Overall, the study did not compromise existing healthcare services and instead contributed positively to the local research and healthcare environment. |
| --- |

1. Confirm that local ethics review was sought, and please provide the approval number. If not sought, please explain why.

| Trial and recruitment materials were approved by the ethics committee at the Hospital Pablo Tobón Uribe, Medellín, Colombia and the Colombian Health Authority, Instituto Nacional de Vigilancia de Medicamentos y Alimentos (ethics approval number PI-BA-796), and monitored by an Independent Data and Safety Monitoring committee. |
| --- |

Secondary analyses

1. Have the data analysed in your study been extracted from another source, such as a national survey, rather than being directly collected by the authors of this paper?

| No |
| --- |

If the authors of this paper were not involved in data collection, how were the findings interpreted with sufficient contextual knowledge?

The Lancet Group *believe contextual understanding is crucial for informed data analysis and interpretation.*

| All authors, regardless of direct participation in data collection, have relevant experience in AD research that enabled them to interpret/review the data from an informed perspective. |
| --- |

1. Please provide the title (eg, Dr/Prof, Mr/Mrs/Ms/Mx), name, and email address of an author who can be contacted about this statement.

| **Name:** Prof. Reiman  **Email:** Eric.Reiman@bannerhealth.com |
| --- |

1. Finally, please provide the title and name of an author from one country of study who has seen and approved this form.

| **Name:** David Aguillon, MD PhD |
| --- |
